# A comparison between mobile and stationary gas chromatography–mass spectrometry devices for analysis of complex volatile profiles

**DOI:** 10.1007/s00216-022-04391-y

**Published:** 2022-11-17

**Authors:** Andrea Marcillo, Juan C. Baca Cabrera, Anja Widdig, Claudia Birkemeyer

**Affiliations:** 1 Research Group of Mass Spectrometry, Institute of Analytical Chemistry, Faculty of Chemistry and Mineralogy, University of Leipzig, Linnéstr. 3, 04103 Leipzig, Germany; 2 Forschungszentrum Jülich GmbH, Institute of Energy and Climate Research (IEK-8), Wilhelm-Johnen-Strasse, Jülich, 52428 Germany; 3 Grassland Group, Technical University of Munich, Alte Akademie 12, 85354 Freising, Germany; 4 Institute of Bio- and Geoscience, Agrosphere (IBG-3), Forschungszentrum Jülich GmbH, Wilhelm-Johnen-Strasse, 52428 Jülich, Germany; 5 Research Group of Behavioral Ecology, Institute of Biology, Faculty of Life Sciences, University of Leipzig, Talstr. 33, 04103 Leipzig, Germany; 6 Research Group of Primate Behavioural Ecology, Department of Human Behaviour, Ecology and Culture, Max-Planck Institute for Evolutionary Anthropology, Deutscher Platz 6, 04103 Leipzig, Germany; 7 German Center for Integrative Biodiversity Research (iDiv), Deutscher Platz 5E, 04103 Leipzig, Germany

**Keywords:** Portable devices, Point-of-care mass spectrometry, Thermal desorption–gas chromatography, Complex VOC analysis, Analytical performance

## Abstract

On-site analysis of volatile organic compounds (VOCs) with miniaturized gas chromatography–mass spectrometry (GC–MS) systems is a very rapidly developing field of application. While, on the one hand, major technological advances are improving the availability of these systems on the market, on the other hand, systematic studies to assess the performance of such instruments are still lacking. To fill this gap, we compared three portable GC–MS devices to a *state-of-the-art* benchtop (stationary) system for analysis of a standard mixture of 18 VOCs. We systematically compared analytical parameters such as the sensitivity and similarity of the signal response pattern and the quality of the obtained mass spectra. We found that the investigated mobile instruments (i) showed *different response profiles* with a generally lower number of identified analytes. Also, (ii) *mass spectral reproducibility* (% relative standard deviation (RSD) of the relative abundance of selective fragments) was generally worse in the mobile devices (mean RSD for all targeted fragments ~9.7% vs. ~3.5% in the stationary system). Furthermore, mobile devices (iii) showed a poorer *mass spectral similarity* to commercial reference library spectra (>20% deviation of fragment ion relative intensity vs. ~10% in the stationary GC–MS), suggesting a less reliable identification of analytes by library search. Indeed, (iv) the performance was *better with higher-mass and/or more abundant fragments*, which should be considered to improve the results of library searches for substance identification. Finally, (v) the estimation of the signal-to-noise ratio (*S/N*) in mobile instruments as a measure of sensitivity revealed a significantly lower performance compared to the benchtop lab equipment (with a ratio among medians of ~8 times lower). Overall, our study reveals not only a poor signal-to-noise ratio and poor reproducibility of the data obtained from mobile instruments, but also unfavorable results with respect to a reliable identification of substances when they are applied for complex mixtures of volatiles.

**Graphical Abstract:**

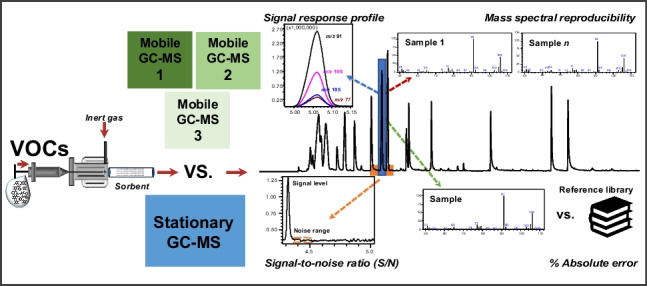

**Supplementary Information:**

The online version contains supplementary material available at 10.1007/s00216-022-04391-y.

## Introduction

Besides quantitative information, mass spectrometry (MS) generates more structural information than many other analytical techniques [1]. The coupling with powerful separation methods such as gas chromatography (GC) substantially improves selectivity even further and allows for analysis with very low detection limits (e.g., for sample amounts in the pg range by capillary GC–MS in benchtop instruments) [2]. Thus, GC–MS still is the method of choice for highly complex samples of sufficient volatility due to its superior selectivity and sensitivity [3]. Its implementation is therefore particularly desirable when rapid and highly accurate identification of substances is especially important [4], such as in mobile analytics.

In time-critical incidents, on-site applications cannot rely on prompt confirmation of results by reference methods [5]. Therefore, mobile analytics in particular requires valid analytical platforms not only providing fast and accurate multi-selective analyses [6], but also enabling easy automation and continuous operation with low maintenance effort [7]. Given the adverse effects of several volatile organic compounds (VOCs) on health and survival of many species including humans (e.g., [8]) and the vast number of possible emission sources in our environment, method development in mobile analytics increasingly focuses on the *simultaneous* analysis of various compounds [9].

Indeed, effective VOC analysis with mobile MS is an important and fast-growing field of research. The advantages of miniaturized systems for on-site applications over laboratory-scale instrumentation are obvious: apart from the immediate availability of the results, analysis consumes very little sample and essential resources, resulting in an ecologically sustainable operation saving energy and raw materials. In addition, these kinds of devices also allow a remote, spatial assessment of many, including inhospitable, environments. Currently, several field-portable GC–MS instruments are commercially available with a wide range of capabilities and limitations [5, 6, 10, 11].

Given the great capacity for detection and identification of chemicals [6], GC–MS has been implemented in portable instruments for different purposes, such as (i) mitigation and safety management assessment (e.g., analysis of chemical warfare agents: [12, 13, –15]), (ii) environmental monitoring (e.g., determination of chlorinated VOCs [16] or munition constituents [17]), (iii) forensic investigations [18], and (iv) in chemical ecology (e.g., for identification of scent components in mammals [5, 10, 19] or volatiles in plants [20, 21]). More recently, portable GC–MS with either thermal desorption (TD) or solid-phase microextraction (SPME) has also been proposed for sampling and analysis of VOCs in human breath [22], as a potential alternative to improve the ability to detect diseases such as COVID-19 infections through potential biomarkers [23].

While many groundbreaking technological developments have advanced laboratory-scale instrumentation at all stages of analysis from sample preparation to sophisticated tools for processing and evaluation, advances in portable GC–MS systems still seem insufficient [24]. Thus, several problems in compound quantification occurred with both, the probe-type [19, 25] and thermal desorption inlets, not always reaching even the minimal requirements of standard procedures for analytical determinations [15, 24]. In studies with individual instruments, a low sensitivity was reported; for instance, for several compounds, the lower limit of quantification could not compete with a GC–MS benchtop instrument [19]. In another study, 13 VOCs also showed higher limits of detection (LOD, in the range of low ppb and ng) with poor accuracy among different portable devices compared to the stationary instrument, e.g., LOD for heptane, 1.19 ppb vs. 0.03 ppb, respectively [14]. Similarly, the analysis of the toxic compound 2,6-dimethylphenol in a portable GC–MS resulted in a higher limit of detection of 7 ppm by direct injection of a liquid solution compared to 1.4 ppm in a benchtop instrument, showing that the sensitivity of the conventional device outperformed the portable device by approximately 5 times [15]. Indeed, due to these differences, comparisons among instruments for the establishment of sensitivity standards have been requested [20]. In addition, analysis was further hampered by poor precision (e.g., normalized relative standard deviation,  RSD across portable devices was between 18 and 42% compared to < 12% with a benchtop system [14]) and low recoveries (e.g., 31 ± 21% by external calibration [24]), requiring different normalization respectively evaluation strategies to improve the reliability of quantitative determinations. For instance, the application of isotopically labeled calibration standards pre-loaded onto TD tubes showed better compensated recoveries with lower deviations, above 90 ± 10% [24]. Therefore, it still takes highly experienced personnel to critically evaluate and process the data obtained with portable TD/GC–MS instruments [6]. Another important shortcoming is the need for widely available standard databases for compound identification in the field [20] and for compound analysis in the background of a complex sample matrix requiring highly selective protocols.

For successful application in the field, portable GC–MS needs to be *fit for the purpose*; a reliable analysis of VOCs becomes only possible by knowing and dealing with the limitations of each instrument [26]. Within this context, we assessed the performance of three commercially available portable GC–MS devices compared to a standard benchtop instrument: (i) the *E*2M with a thermal desorption (TD) unit (Bruker), (ii) the *H*apsite ER with a TD unit (Inficon), and (iii) the *T*orion T-9 with a solid-phase microextraction (SPME) inlet (PerkinElmer). From now on, these portable GC–MS instruments will be referred to herein as “MobE” (i), “MobH” (ii), and “MobT” (iii), respectively, and the conventional TD/GC–MS instrument as “Stationary.” We analyzed a complex standard mixture of VOCs to identify performance limits of these devices hampering high-quality measurements, and to suggest how to improve the quality of on-site GC–MS analysis of organic compounds in unknown volatile profiles.

## Experimental

### Materials and chemicals

Empty and pre-cleaned thermal desorption (TD) glass tubes (6.35 mm O.D. × 89 mm length) and Tenax TA Porous Polymer (Tenax TA, 60–80 mesh) were purchased from Supelco/Sigma-Aldrich (Taufkirchen, Germany) and assembled in-house as described before [27], following the guidelines of the U.S. Environmental Protection Agency (EPA) in the *Compendium Method TO-17 Determination of Volatile Organic Compounds in Ambient Air Using Active Sampling onto Sorbent Tubes* [28]. Pre-assembled and larger TD tubes (8 mm O.D. × 110 mm length) filled with Tenax TA (60–80 mesh) were purchased from Günther Karl OHG (Gau-Algesheim, Germany). For conditioning of standard tubes, a TD Clean Cube unit (SIM Scientific Instruments Manufacturer GmbH, Oberhausen, Germany) flushed with nitrogen from a generator (Nitrox UHPLCMS 18 Domnick Hunter, Gateshead, UK) was used. For larger tubes, a GC oven (dismantled from a 5890 GCMS, Agilent, Santa Clara, USA) was in-house adapted for constant flow of nitrogen carrier gas and a ramped temperature program for conditioning. Exceptionally, for the MobT, 65-µm pre-conditioned PDMS-DVB fibers assembled to a SPME syringe (PerkinElmer, Germany) were used for sample introduction.

A microsyringe (Hamilton CO, USA) was used for loading 1 µL of the liquid standard mixture onto the non-sampling end of the thermal desorption tubes (two sizes) at a nominal flow rate of 100 mL/min nitrogen and adjusted by a *CFC-14PM* multichannel gas flow regulator for *GC-14A* GC–MS (Shimadzu, Kyoto, Japan).

Methanol (solvent, HiPerSolv Chromanorm) was purchased from VWR International S.A.S., USA. Acetone p.a. ≥ 99.8% (SupraSolv® for GC–MS), diethylamine (for synthesis), and xylene (*o*-xylene) were purchased from VWR International GmbH (Darmstadt, Germany). Chloroform (for liquid chromatography), *n*-hexane (SupraSolv for GC), and toluene (SupraSolv) were purchased from Merck (Darmstadt, Germany). 2-butanol p.a. 99% was purchased from Sigma-Aldrich (St. Louis, USA). Pyridine p.a. ≥ 99.8% was purchased from Sigma-Aldrich (Taufkirchen, Germany). Cyclohexane p.a. ≥ 99.9% was purchased from Carl Roth GmbH & Co. KG (Karlsruhe, Germany). *n*-Nonane was purchased from Promochem (Wesel, Germany). Phenol was obtained from Novapex, Saint Maurice L’Exil, Cédex, France. Pentafluorobenzene p.a. 98% was purchased from Alfa Aesar GmbH & Co KG (Karlsruhe, Germany). Aniline p.a. 99.8% was purchased from Acros Organics – Fisher Scientific GmbH (Schwerte, Germany). 4-Chlorophenol and 3,4-dichlorophenol were purchased from Riedel-de Häen (Seelze, Germany). 2,4,5-Trichlorophenol p.a. ≥ 99% was purchased from Fluka (Neu-Ulm, Germany). Benzene and 1-hexanol were purchased from other vendors.

### Preparation of volatile standard mixtures and samples

The preparation protocol was adapted from the procedure recommended by the U.S. Environmental Protection Agency (EPA), Method 8260 C Volatile Organic Compounds by Gas Chromatography/Mass Spectrometry (GC–MS) [29]. A liquid standard mixture of 18 volatile compounds in methanol was prepared for the evaluation of all devices. The composition of the standard mixture accounted for a broad range of volatility to cover a large elution range and the presence of several chemical classes to mimic potential variability in unknown samples. Stock solutions of authentic standards were prepared at 100 mM by weighing and adding the pure standard directly to the required volume of solvent. Afterwards, secondary dilutions were prepared to achieve mixtures of similar concentration by mixing specific volumes of each standard solution in methanol. Stock solutions and their corresponding secondary dilutions were freshly prepared before each set of experiments. Table 1 shows the composition of each standard mixture at the concentration range used for all devices.

**Table 1:** Composition of the standard mixture (18 compounds) organized by elution order at high concentrations according to the specific upper part of the linear range of the corresponding compound in the stationary device using Tenax TA, listed for each GC–MS device

No	Compound	Predicted b.p. [°C]^a^	Molecular weight [g/mol]^a^	Concentration [mM per compound in solution] │ [ng of compound per tube]							
				Stationary^b^		MobE		MobH^b^		MobT^b^	
1	Propan-2-one	46 ± 3	58	1.0	56	0.9	51	1.0	56	1.0	–
2	*N*-Ethylethanamine	57 ± 8	73	0.9	66	0.9	68	0.9	66	0.9	–
3	Hexane	68 ± 3	86	1.0	85	0.9	75	1.0	85	1.0	–
4	Butan-2-ol	97 ± 3	74	1.0	72	0.9	69	1.0	72	1.0	–
5	Chloroform	61 ± 8	119	0.9	111	0.9	110	0.9	111	0.9	–
6	1,2,3,4,5-Pentafluorobenzene	86 ± 35	168	0.8	141	1.0	166	0.8	141	0.8	–
7	Benzene	79 ± 7	78	1.0	79	1.0	78	1.0	79	1.0	–
8	Cyclohexane	81 ± 0	84	1.0	84	0.8	68	1.0	84	1.0	–
9	Pyridine	115 ± 0	79	1.0	78	1.0	75	1.0	78	1.0	–
10	Toluene	111 ± 3	92	1.0	90	1.0	92	1.0	90	1.0	–
11	Hexan-1-ol	158 ± 3	102	1.0	105	1.0	100	1.0	105	1.0	–
12	1,2-Xylene	146 ± 10	106	1.0	105	0.9	96	1.0	105	1.0	–
13	Nonane	152 ± 3	128	0.9	117	0.9	121	0.9	117	0.9	–
14	Aniline	184 ± 0	93	1.0	93	1.0	91	1.0	93	1.0	–
15	Phenol	182 ± 0	94	1.0	89	1.0	92	1.0	89	1.0	–
16	4-Chlorophenol	220 ± 0	128	0.9	120	1.0	131	0.9	120	0.9	–
17	2,4,5-Trichlorophenol	255 ± 35	197	1.0	188	1.0	205	1.0	188	1.0	–
18	3,4-Dichlorophenol	247 ± 20	163	1.0	170	1.1	174	1.0	170	1.0	–
Minimum				0.8	56	0.8	51	0.8	56	0.8	–
Maximum				1.0	188	1.1	205	1.0	188	1.0	–

Boiling points were retrieved in January 2020 from the public database SciFinder – CAS (SciFinder – CAS)

*No.* designated ID number for each compound, *b.p.* boiling point, – values not added for comparison because a different introduction device and extraction methods were used

^a^Predicted boiling points at standard pressure (760 Torr) and monoisotopic molecular weights were retrieved from SciFinder – CAS

^b^Same solution for Stationary, MobH, and MobT

Due to the poor sensitivity for mobile devices obtained in our first tests (data not shown), a high-level concentration range was selected for the comparison, matching the highest tested concentration of the linear range in the Stationary [27, 30]. Almost all compounds (except benzene and hexanol) were already evaluated for adsorption to Tenax TA with subsequent analysis by stationary TD/GC–MS in terms of sensitivity (lower limits of quantitation, LLOQs), linearity, reproducibility, relative recovery, and breakthrough values in a range between 0.01 and ~ 250 ng/tube. Identical conditions were used to prepare spiked TD tubes: 1 µL of the corresponding serial dilution was injected into a constant nitrogen flow of 100 mL/min to carry the analytes through the sorbent bed of Tenax TA during 13 min of collection time. For conditioning of standard thermal desorption tubes (6.35 mm O.D. × 89 mm length), a TD Clean Cube unit (SIM Scientific Instruments Manufacturer GmbH, Oberhausen, Germany) was used flushed with nitrogen from a generator (Nitrox UHPLCMS 18 Domnick Hunter, Gateshead, UK). For larger thermal desorption tubes (8 mm O.D. × 110 mm length), a GC oven (5890 GCMS, Agilent, Santa Clara, USA) was adapted for heating under a ramped temperature program and inert nitrogen gas flow.

### Experimental design and instrumental parameters of analysis on mobile TD/GC–MS instruments

Three mobile GC–MS devices were evaluated and compared to a *state-of-the-art* benchtop stationary GC–MS, labeled here as *Stationary* (TD-20 thermodesorber with GC 2010 plus TQ8040 MS, Shimadzu, Kyoto, Japan). The following models were used: (i) the E2M with a TD unit (Bruker Daltonics GmbH, Leipzig, Germany), *MobE*; (ii) the Hapsite ER with a TD unit (INFICON Holding AG, Switzerland), *MobH*; and (iii) the Torion T-9 with an SPME inlet (PerkinElmer, USA), *MobT*. The optimized instrumental parameters for the comparison among the devices are described in detail in the electronic supplementary material (Supp. S.1).

Prior method optimization was required due to the specific differences in the instrumental configuration of each device including the introduction of the samples. In general, two introduction devices, TD and SPME, were employed to test their suitability for analysis of complex mixtures of VOCs in mobile GC–MS systems. TD was selected since it ensures a known amount of sample to be introduced to the analysis in comparison to probe sampling. The sampling was based on the adsorption to a similar sorbent material across the instruments and subsequent desorption by high temperature. For MobT, however, TD was not available and SPME was used instead.

#### Stationary

Analysis was carried out as described in previous works [27, 30], summarized in the supplementary section (Supp. S.1). Briefly, the standard-spiked TD tubes were desorbed at 250 °C and the compounds subsequently separated by GC using a linear temperature gradient.

In addition, a *GCMS-QP2010* system composed of a gas chromatograph *GC-2010 Plus* coupled to a quadrupole mass spectrometer (Shimadzu, Kyoto, Japan) was used as a second non-mobile/lab-installed GC–MS device to estimate the reproducibility of the mass spectra under different vacuum conditions only. The samples were introduced into the instrument by a head space system *HS-20* (Shimadzu, Kyoto, Japan) by both trap and loop mode and labeled as “Stat HS-TM” and “Stat HS-LM,” respectively. For further detail on the applied instrumental parameters, refer to Supp. S.2, Table S.2.1.

#### MobE

Chromatographic separation of fast-eluting compounds was considerably improved by a low slope (5 K/min) at the beginning of the temperature program. However, before reaching the highest temperature, faster ramping (10 K/min) successfully reduced the elution time of the semi-volatile chlorinated phenols without considerably changing the resolution (i.e., peak widths were barely reduced) and intensity of the chromatographic peaks.

#### MobH

Between two methods different by the starting temperatures of membrane, valve oven and heated lines (120 °C and 110 °C instead of 80 °C and 70 °C) and the thermal desorber (290 °C instead of 250 °C), the high-temperature program was finally selected since it considerably improved the molar response of most of the compounds along the entire range of volatility, improving the signal response of the semi-volatile chlorinated phenols and several early-eluting compounds (very volatile compounds VVOCs), from which the latter were probably lost due to potential adsorption during the transfer between the thermal desorber and the chromatographic column with the low-temperature method. Nevertheless, the high temperature method also enhanced the signal response of the compounds eluting in the middle range of volatility, and even saturation occurred (i.e., 1,2-xylene, nonane, phenol, and 4-chlorophenol).

Two additional portable devices of the same type as MobH but different introduction devices and labeled accordingly (i.e., *MobH-SPME* and *MobH-Sample Probe*) were used only for estimation of mass spectral reproducibility at different high vacuum pressures and applying the already described high-temperature method without occurrence of saturation (refer to Supp. S.2, Table S.2.1 for an extended description of the applied instrumental parameters).

#### MobT

A PDMS/DVB SPME syringe was used for sampling the headspace (HS) of a closed glass vial of 9.1 mL total volume, containing a specific volume of standard mixture in methanol (spiked directly in the vial as in [31]), at variable extraction times (0.5, 1, 5, and 30 min), desorption times (5 and 30 s), and sample gas volumes (splitless, split 10:1 and 50:1), and hereafter labeled as *MobT HS*. Since the extraction time for mixtures can be determined by the time required for the slowest compound to reach the equilibrium (equilibration time) [32], we tested different extraction times but found no differences in the signal responses between 1 and 5 min for the slowest compound in our experiment (1,2-xylene). With the largest extraction time at 30 min, we did not detect any other compound with lower volatility or slower than 1,2-xylene; thus, we used for this experiment the lowest tested extraction time. In addition, the direct immersion (DI) of the fiber into the standard solution for 0.5 min extraction time and 5 s desorption time was also tested (denoted hereafter *MobT DI*) and resulted in a different signal response profile with a larger number of identified compounds, especially the ones with lower volatility and higher polarity (as expected from the direct immersion extraction mode), such as the chlorinated phenols (supplementary section, Supp. S.3). However, the data from MobT (HS and DI) was used only for the comparison of mass spectral reproducibility and similarity since different sampling procedures should not result in a higher variance of mass spectra themselves; it could not be applied for comparison of the signal response pattern and signal-to-noise ratio (except for searching trends).

### Data evaluation

GCMS solution 4.20 software (Shimadzu, Kyoto, Japan) was used for integration of the signals of all identified compounds in three devices but not MobH, which raw data could not be converted into a suitable data format. Instead, a conversion tool of ER IQ software version 2.33 “.hps” to “.csv” files (Inficon, Inc., Germany) was used to export the total ion current (*m/z* 45–300, total scan number 1063). Then, areas of selective mass traces in the extracted ion chromatograms (XIC) were calculated manually as the sum of areas over the retention time range between the peak start and end of each identified analyte.

Four mass traces were chosen per analyte, i.e., one ion used for quantitation (denoted as “*quan* ion” and labeled as “*m/z*_1_”) and three identity-confirming selective (“qualifier”) ions, labeled as “*m/z*_2_,” “*m/z*_3_,” and “*m/z*_4_” (Supp. S.4) and organized by descending relative abundance in the mass spectrum. Relative abundances of selective mass traces of standard spectra were retrieved from the MAINLIB of the NIST/EPA/NIH 14 mass spectral library (National Institute of Standards and Technology, Gaithersburg, MD, USA). Data normalization and most calculations were performed using Microsoft Excel 365 (Microsoft Corp., Redmont, USA).

#### Data normalization

A great challenge in comparing such different instrumentation and raw data was to find the most appropriate methodology for analysis and strategies of data normalization. We developed different normalization strategies depending on the analytical parameter to be evaluated. Thus, for *response pattern comparison*, data was *maximum-normalized* to the most abundant signal among all compounds within a chromatogram to unify a scaling over different orders of magnitudes for absolute intensity among devices. *Normalization of the abundance of selective ions to the quan ion* was employed to assess the mass spectral precision in terms of *reproducibility of the mass spectral pattern*. Finally, *normalization of fragment intensity to the corresponding value in the NIST reference spectrum* was employed to assess the accuracy of the obtained spectra, denoted as *mass spectral similarity*.

*Maximum normalization*, i.e., division of each *quan ion* area (*m/z*_*1*_ of each compound of each replicate) by the mean area of replicate analysis of the *quan ion* of the most abundant signal among all compounds from given data set of each individual instrument (i.e., the most intense analyte signal from the standard mix detected with a particular instrument equals 1 or 100%, respectively), brought each signal response pattern to scale for convenient comparison among devices (labeled as “*maximum-normalized response*”). Maximum normalization was also applied to compare the fragment ion abundances across all analytes measured with a particular instrument. For this, the height of the chromatographic peak apex of each ion was divided by the one with the largest height across all compounds detected with a particular instrument.

*Normalization to the quan ion (m/z*_*1*_*)* was applied to eliminate the differences in absolute intensity of *quan* and identity-confirming ions among devices and replicates (a common way to present EI spectra using the relative ion abundance after normalization to the highest, “base” peak of a given spectrum), to assess the variability in the *relative* abundance of each selective mass trace in a mass spectrum, i.e., the reproducibility of the *fragment pattern* (*mass spectral reproducibility*). For this purpose, the height of the chromatographic peak at the apex of each selective ion (three qualifier and one *quan* ion per analyte and replicate) of a given analyte was normalized to the height of the base peak at the apex (*quan ion*, *m/z*_*1*_) expressed in percentage (labeled as *“relative abundance”*). The relative standard deviation (% RSD) was calculated from the resulting relative abundances for all replicate analyses (per ion, analyte, and device).

*Normalization to the corresponding reference value* was applied to estimate how well the relative abundance of each selective mass trace matches the fragment pattern of the reference mass spectrum from the MAINLIB of the NIST/EPA/NIH 14 mass spectral library. Therefore, the “relative abundances” (as calculated before for mass spectral reproducibility) were related to the corresponding relative abundance of the same fragment (division of the *observed* by the *expected* value of the same fragment in the reference spectrum), and finally labeled as “normalized ratio.” In addition, the “normalized ratio” minus one was labeled as the “absolute error” and expressed in percentage. For library search, the mass spectrum is normally calculated from selected regions of the chromatographic peak, e.g., as average spectrum around the apex, eventually followed by background subtraction. Here, the reproducibility of the mass spectra was estimated through the height of the chromatographic peak (i.e., intensity at the peak apex minus the noise) as an equivalent to a background-subtracted MS at the apex. This let us avoid the contribution to the variance of poorly resolved peaks, asymmetry and coelution for some of the detected analytes. Since for MobH, the conversion of the raw data was not feasible, reproducibility and similarity of the mass spectra were estimated through the intensity of the scan at the apex of the peak, except on those saturated peaks, on which a scan free of saturation was chosen.

#### Calculation of the signal-to-noise ratio (*S/N*) for the analyzed compounds

The signal-to-noise ratio (*S/N*) for each instrument was estimated by automatic computing using the *GCMS solution* 4.20 software (Shimadzu, Kyoto, Japan) of the *Stationary* to ensure an accurate comparison. (Note that MobH was excluded since the data could not be reasonably exported from the acquisition software.) According to the supplier’s specifications [33], the *signal level* was calculated through the difference between the maximum intensity within the peak detection time range (*signal range*) and the average intensity corresponding to the noise detection range (*noise range*). The root-mean-square (RMS) method was used for calculation. The noise was obtained as the standard deviation of the single signals identified in a *noise range* of 1.5 min (approximately 10 times the peak width, e.g., for 1,2-xylene in the stationary system). For MobE and MobT, the noise ranges were similarly set according to the performance of 1,2-xylene: 1.5 min (1.5 times the peak width) and 0.3 min (around 10 times the peak width), respectively. A noise range of 10 times the peak width for MobE was not feasible due to rather broad peaks; therefore, the *S/N* was tested along all the integration time range (the maximal possible value of 3 times the peak width) with no differences in the corresponding values. *S/N* was calculated for four selective mass traces of each compound identified in analyses from the Stationary, MobE, and MobT. For Stationary and MobE only, analytes (*n*_analyte_ = 13) identified with both instruments were considered for comparison between each other; in addition, 2,4,5-trichlorophenol and 3,4-dichlorophenol were excluded due to bad reproducibility on MobE.

#### Statistical analysis

Statistical analysis was carried out using the software R (version 4.0.3) [34], and the ggplot2 package [35] was used for data plotting. We assessed differences of our results regarding (i) the *signal response pattern* of the maximum-normalized response of all detected analytes across the instruments, (ii) the *mass spectral reproducibility* as % RSD of the relative abundance of each target ion in replicate analyses, (iii) the *mass spectral similarity* as the % absolute error against the reference value in the commercial mass spectral library NIST14, and (iv) the *sensitivity* as the signal-to-noise ratio (*S/N*) of *quan* and/or confirming ions. Statistical analyses for the comparison were the following:

1. A series of non-parametric Mann–Whitney *U* tests between each portable device and the reference system was applied for the following variables: % RSD, % absolute error, and *S/N*, to achieve a general comparison of the different GC–MS devices (refer to “General performance of the different GC–MS devices for VOC analysis”). As a result, the mean of each variable, the corresponding standard error, and the significance (defined as *p* value < 0.05) are presented in Table 2. No normalization was required for this analysis.
2. An ANOVA was used to test the effect of the boiling point on the maximum-normalized response for each device (Stationary, MobE, and MobH; refer to “Signal response patterns of the VOC standard mixture after TD/GC–MS analysis differ between the evaluated instruments”). The tabulated results can be found in the supplementary section (Supp. S.5).

**Table 2:** General comparison of each portable device, MobE, MobH, and MobT, to the reference (Stationary) in terms of *mass spectral reproducibility* (% RSD of the relative abundance in replicate analysis per selective ion), *mass spectral similarity* (% absolute error: absolute value of the “normalized ratio” minus one and expressed in percentage), and *signal-to-noise ratio* (*S/N* per selective ion by RMS). A series of non-parametric Mann–Whitney *U* tests reported significant differences between each portable device (mean ± standard error (SE)) and the reference system (mean ± SE), labelled in bold. Significance was defined as *p* value < 0.05

Analytical parameter	Unit of measure	Parameter	Stationary	MobE	MobH	MobT
Mass spectral reproducibility	% RSD (observed relative abundance in replicate measurements)	Mean ± SE	3.5 ± 0.4	**10.9 ± 1.5**	5.3 ± 0.9	**12.6 ± 1.3**
		*p* value	–	** < *0.001***	*0.07*	** < *0.001***
		(*n*_analyte_, *n*_ion_, *n*_sample_)	(17, 50, 7)	(13, 36, 3)	(15, 41, 9)	(6, 18, 17)^b^, (9, 27, 2)^c^
Mass spectral similarity	% absolute error	Mean ± SE	21.6 ± 4.3	23.5 ± 3.3	**37.6 ± 6.9**	**38.2 ± 4.5**
		*p* value	–	*0.053*	** < *0.01***	** < *0.001***
		(*n*_analyte_, *n*_ion_, *n*_sample_)	(17, 49, 7)	(13, 33, 3)	(15, 37, 9)	(6, 18, 17)^b^, (9, 27, 2)^c^
Sensitivity	(Signal/noise by RMS method)	Mean ± SE	6340 ± 2320	**119 ± 26**	n.a.^a^	**556 ± 135**^**b**^
		*p* value	–	** < *0.001***		** < *0.001***
		(*n*_analyte_, *n*_ion_, *n*_sample_)	(11, 44, 7)	(11, 36, 3)		(5, 20, 17)^c^, (7, 28, 2)^d^

For further details related to c and d, refer to “Experimental design and instrumental parameters of analysis on mobile TD/GC–MS instruments” and Supp. S.1

*n*_*analyte*_ number of identified analytes, *n*_*ion*_ number of selective ions from all identified analytes, *n*_*sample*_ number of replicates, *n.a.* values not available

^a^*S/N* could not be estimated by using GC–MS solution software as for the other instruments

^b^*S/N* values for MobT cannot be compared because a different injection system and sorbent material were used

^c^Detected compounds using HS-SPME with a PDMS-DVB fiber, MobT HS

^d^Detected compounds using DI-SPME with a PDMS-DVB fiber, MobT DI

Ordinary least squares regression analysis was used to evaluate the effect of the explanatory variables: (i) *m/z* (= mass) of the fragment, (ii) a fragment’s abundance as the height of the peak at the apex of each ion was divided by the one with the largest height across all compounds detected with a particular instrument, and their interaction on the following *response variables*: (i) % RSD (refer to “Mobile instruments exhibit a poorer mass spectral reproducibility”); (ii) % absolute error (refer to “Mobile instruments exhibit a poorer mass spectral similarity for identification with mass spectral libraries”); and (iii) *S/N* (refer to results section “Mobile instruments exhibit a lower sensitivity”) for each instrument individually. Normal distribution of each log-transformed variable was tested with a Lilliefors (Kolmogorov–Smirnov) normality test, and the results showed normal distribution in all cases (*p* value > 0.05). Results from the regression analysis are presented in terms of intercept (*b*), slope (*m*), and significance (defined as *p* value < 0.05) in the supplementary section (Supp. S.6).

## Results and discussion

### General performance of the different GC–MS devices for VOC analysis

Using a series of non-parametric Mann–Whitney *U* tests, significant differences were found for mean values of mass spectral reproducibility, mass spectral similarity, and signal-to-noise ratio (as a measure of sensitivity) between each portable device (MobE, MobH, and MobT) and the reference system (Stationary), indicating a limited performance for analysis of complex volatile profiles. The results are presented in Table 2.

Mass spectral reproducibility was significantly worse for MobE and MobT (both *p* values < 0.001) but less for MobH (*p* value = 0.07) in comparison to the Stationary; i.e., mass spectra obtained from the conventional GC–MS were more reproducible (lower % RSD) than from any of the evaluated portable instruments independent of the mass analyzer (quadrupole or toroidal ion trap) and/or introduction device (thermal desorption or SPME). In addition, mass spectral similarity with the NIST reference library was also significantly lower with two out of three portable devices with different mass analyzers, MobH and MobT, and, interestingly, the mass spectral match of selective fragments in MobE was only marginally lower (*p* value = 0.053) than that of the Stationary. Both results imply that the qualitative (mass spectral comparison with MAINLIB of the NIST/EPA/NIH library) and quantitative analyses by selective mass traces seem not yet to keep up with the *state of the art* of modern GC–MS.

Furthermore, the *S/N* showed considerably lower mean values for the portable TD/GC–MS systems compared to the reference instrument, which suggests a limited sensitivity of mobile devices in the analysis of complex VOC mixtures. (Note that as already mentioned in experimental section “Experimental design and instrumental parameters of analysis on mobile TD/GC–MS instruments”, *S/N* values for MobT could not be compared because of a different injection system and sorbent material, which might well change the recovery of compounds and the sorbent background.) Therefore, in the following, we searched our data in more detail for possible determinants of the lower performance of the portable instruments.

### Detailed performance differences between stationary and mobile instruments

#### Signal response patterns of the VOC standard mixture after TD/GC–MS analysis differ between the evaluated instruments

**Fig. 1:**
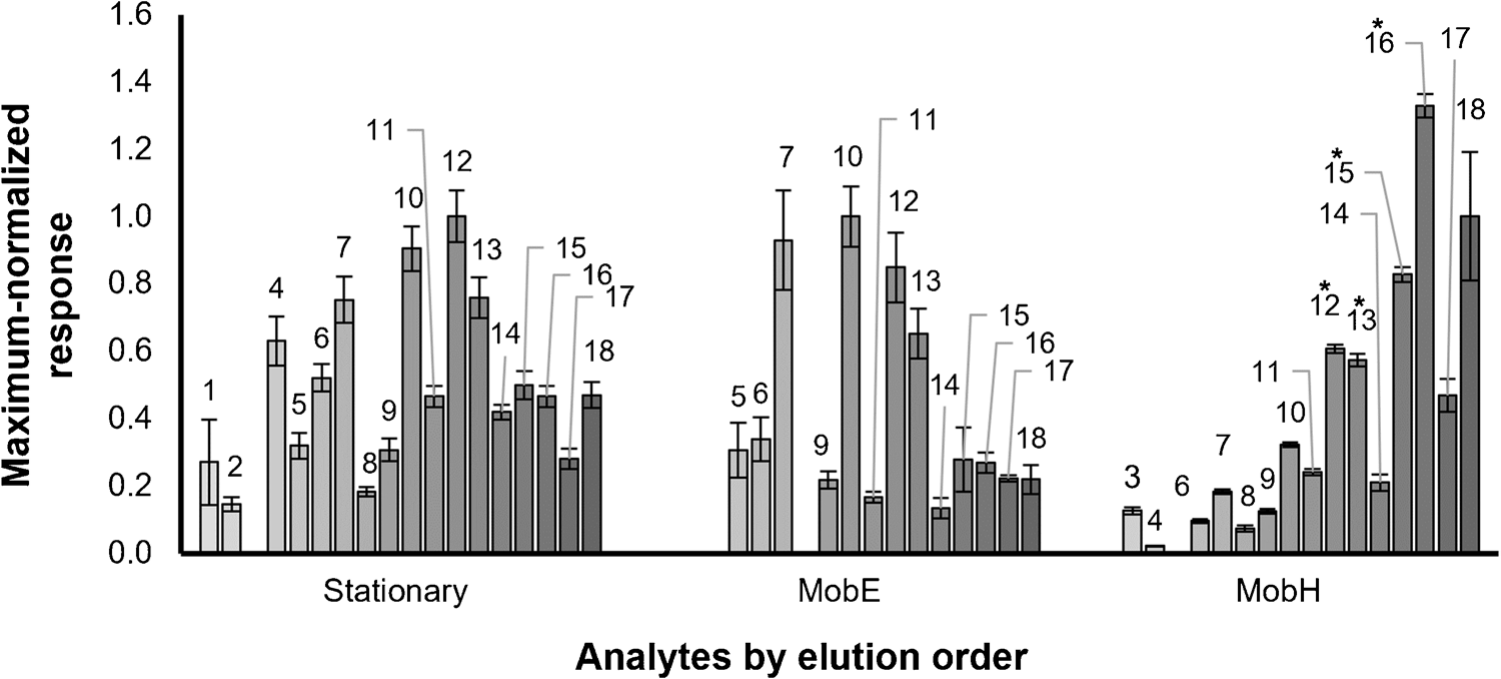
Maximum-normalized response of the standard mixture of 18 compounds (for identification of labeled numbers and further details on detected compounds, refer to Table 1 and Supp. S.4) analyzed on TD/GC–MS Stationary and mobile devices. The integrated areas (each replicate) from the *quan ion* (*m/z*_*1*_) of all identified analytes in the standard mixture were normalized to the mean area of the *quan ion* with the maximum value among all compounds (labeled as “*maximum-normalized response*”). 1,2-Xylene (12), toluene (10), and 3,4-dichlorophenol (18) were used for maximum normalization for the Stationary, MobE, and MobH devices, respectively. The compounds detected with the Stationary (*n*_analyte_ = 17, *n*_sample_ = 7), MobE (*n*_analyte_ = 13, *n*_sample_ = 3), and MobH (*n*_analyte_ = 15, *n*_sample_ = 9, 4 out of 15 compounds were saturated, i.e. No. 12, 13, 15, 16 labelled with an asterisk) were organized and colored (light to dark gray) according to the elution order in the stationary system. Terms’ labels: “*n*_analyte_” = number of identified compounds and “*n*_sample_” = number of replicates

Figure 1 shows the profile of signal responses after normalization of each *quan ion* (*m/z*_*1*_) to the mean area of the most abundant *quan ion* among all analyzed compounds (maximum normalization) after TD/GC–MS analysis of the VOC standard mix with the Stationary (reference) compared to the two portable quadrupole instruments, MobE and MobH. Analytes are organized by elution order in the Stationary.

The response pattern from the Stationary seems more similar to MobE, while MobH had a lower molar response for the early-eluting compounds. Moreover, considerable differences in the number of identified analytes (*n*_analyte_) were found among instruments: 17 (*n*_sample_ = 7), 13 (*n*_sample_ = 3), and 15 (*n*_sample_ = 9) out of 18 compounds in the standard mixture for the Stationary, MobE, and MobH, respectively. The largest number of analytes was recovered with the conventional device; indeed, the lower performance of portable GC–MS devices was expected since preliminary experiments with these instruments (not shown) already indicated several problems in the recovery of very volatile organic compounds (VVOCs and VOCs), most likely related with coelution and peak broadening. Also, other studies have shown a lower number of identified compounds with MobE and MobH (e.g., [5, 19]) in gaseous samples compared to stationary devices. Unfortunately, different stationary and mobile phases (column dimensions and materials, and various carrier gases; see Supp. S.1) tightly configured with the commercial mobile devices could not be changed to improve chromatographic separation or enable a complete orthogonal comparison, and it cannot be excluded that the variability in the resulting compound profiles might at least partly be related to these differences in the chromatographic system. A more flexible instrument configuration and the availability of more stationary phases would not only improve the comparability between instruments but also allow for optimization of chromatographic resolution.

**Fig. 2:**
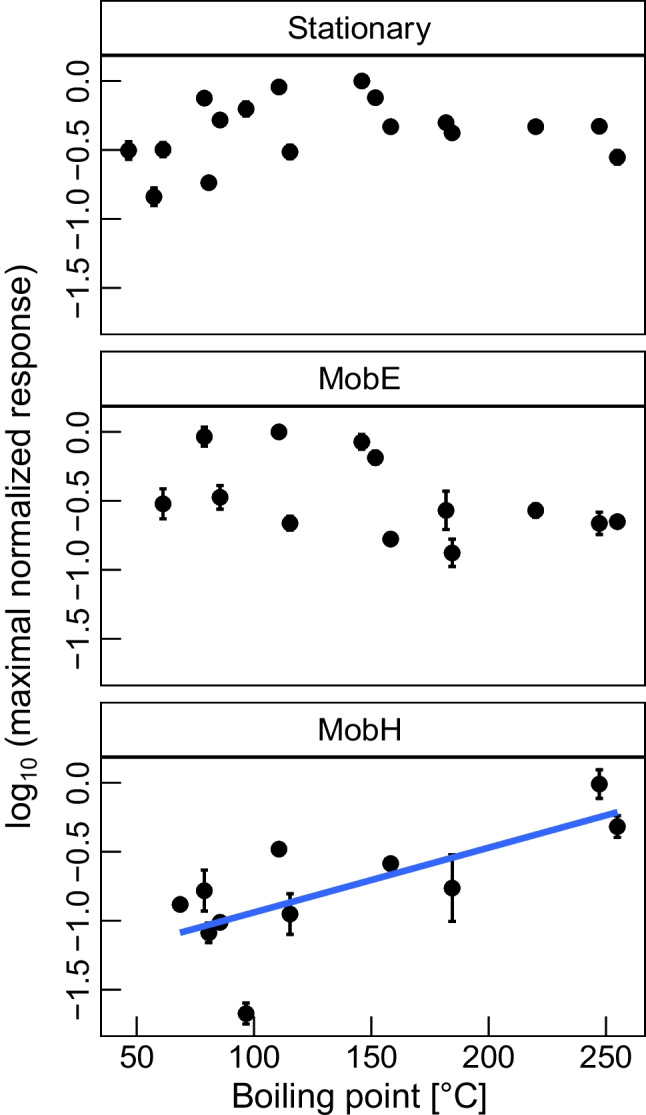
Maximum-normalized response (logarithmic scale) of the *quan ion* of all identified analytes of the stationary device (*n*_analyte_ = 17, *n*_sample_ = 7) and the two portable systems (*n*_analyte_ = 13, *n*_sample_ = 3 and *n*_analyte_ = 11, *n*_sample_ = 9 for MobE and MobH, respectively) in dependence on increasing boiling point. Terms’ labels: “*n*_analyte_” = number of identified compounds and “*n*_sample_” = number of replicates. The blue line represents a significant linear correlation

Moreover, as already seen for all devices (refer to Fig. 1), the maximum integrated area among all analytes in our standard mixture came from compounds with different volatility (e.g., 1,2-xylene (12), toluene (10), and 3,4-dichlorophenol (18) in the Stationary, MobE, and MobH devices, respectively). Therefore, we evaluated the relative response of the compounds in dependence on the boiling points (as a proxy of volatility). The encountered differences between stationary and portable devices are represented through a ggplot in Fig. 2.

According to the results illustrated in Fig. 2 and the corresponding ANOVA (refer to Supp. S.5), we found that the relative signal response of the *quan* ions (i.e., base peaks) of the early-eluting compounds (mostly non-aromatic) in our standard mixture increased with higher boiling points for MobH. The high-temperature program improved the desorption of compounds with lower volatility (between VOCs and SVOCs), such as the chlorinated phenols, and considerably increased the signal response of most of the compounds along the entire range of volatility. The results in Fig. 2, however, suggest a recovery loss of very volatile compounds (probably lost due to adsorption during transfer from the desorber to the chromatographic column in the low-temperature method), and even some of them (e.g., the early-eluting propan-2-one and chloroform) were not detected at all.

This suggests that a more sensitive quantitation in the portable devices by desorption at higher temperatures occurs for compounds with higher boiling points by better desorption and compound transfer (data not shown), and better peak widths are achieved by increasing the heating ramps during chromatographic separation (e.g., in MobE) rather than by optimizing the chromatographic phases (e.g., stationary phases and various mobile phases). Such efforts, however, would have to compromise on the heat stability of the targeted analytes and durability of the instrument’s vacuum connections. Considering the simultaneous loss of compounds with higher volatility, it seems that compound transfer on the mobile devices in particular still requires crucial technical improvements.

As a final remark, we would like to refer the interested reader to the supplementary section (Supp. S.3) for a comparison including MobT, which was excluded here from this comparison because a thermal desorption device was not available for this instrument. Other studies also showed differences in the number of identified analytes and the response profiles of VOCs [36] using the Torion T-9 portable GC–MS [10, 21]. A lower number of identified VOCs with the portable device due to a weak chromatographic resolution and increased coelution [21, 37] was related to the rapid temperature program and shorth length of the column in comparison with the benchtop GC–MS system. These results somewhat agree with the general lower sensitivity of the other mobile instruments (MobE and MobH), most likely also related with peak broadening and coelution.

#### Mobile instruments exhibit a poorer mass spectral reproducibility

The simultaneous introduction of reference standards with the sample itself (“co-spiking”) is usually considered a final confirmation of a substance’s identity; nevertheless, such standards are often not available in the laboratory, and the situation becomes even more difficult during applications in the field. Consequently, obtaining (EI) spectra as a reproducible fingerprint of the target compound and as similar as possible to the reference library is a particularly important feature to achieve a high reliability for library identification when co-injection is not feasible.

Therefore, we examined the reproducibility of the obtained spectra as a first criterion for the reproducibility of compound identification based on spectral comparison with any mass spectral library. It is known that the variance plotted along the dynamic range of the intensity from an analytical detector (here: variance over fragment *intensity*) often exhibits a “trumpet” shape (heteroscedasticity), meaning that the variance is higher to the lower end of the dynamic range and improves toward the middle part or upper end of the dynamic range. We assessed the extent of this appearance with the different instruments. Table 3 presents the relative standard deviation (% RSD) of the *relative abundance* of replicate analyses of three selective mass traces per analyte, categorized in two ranges: *a*_1_ ≤ 25% < *a*_2_ (where *a* is the relative abundance in % and *n*_ion_, the number of selective ions from all identified analytes in that particular abundance range)*.*

**Table 3:**
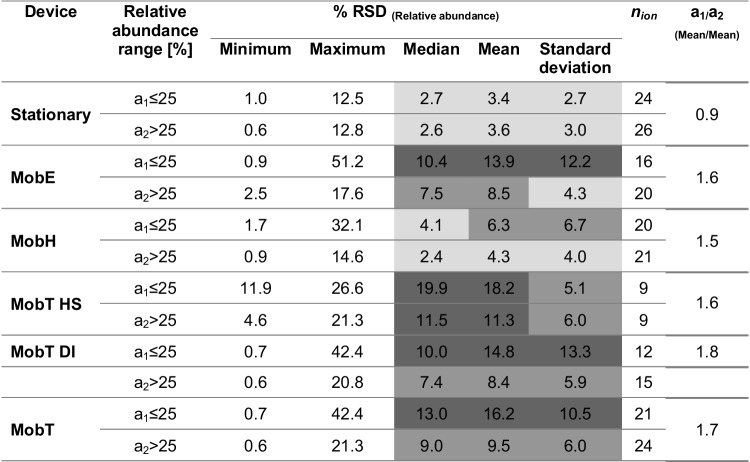
Relative standard deviation (% RSD) of the relative abundance of replicate analysis of three fragments per analyte in two ranges (*a*_1_ ≤ 25% < *a*_2_) for the Stationary (*n*_analyte_ = 17, *n*_ion_ = 50, *n*_sample_ = 7, except for butan-2-ol *m/z* 43) and the portable systems: MobE (*n*_analyte_ = 13, *n*_ion_ = 36, *n*_sample_ = 3, except for pyridine *m/z* 78, 51 and aniline *m/z* 65), MobH (*n*_analyte_ = 15, *n*_ion_ = 41, *n*_sample_ = 9, except for butan-2-ol *m/z* 43 and pyridine), MobT HS (*n*_analyte_ = 6, *n*_ion_ = 18, *n*_sample_ = 17), and MobT DI (*n*_analyte_ = 9, *n*_ion_ = 27, *n*_sample_ = 2). Note that a few *m/z* with insufficient selectivity were excluded from evaluation

Color scheme: light grey for favorable (RSD < 5%), grey for neutral (5 < RSD < 10%), and dark grey for less favorable values (RSD > 10%) per category defined in each column label

*a* relative abundance, *n*_*analyte*_ number of identified analytes, *n*_*ion*_ number of selective fragments of all identified analytes, *n*_*sample*_ number of replicates

In general, the best reproducibility (lower % RSD, colored in light gray) was obtained with the Stationary across the entire range of relative abundance (~ 3.5% mean RSD, *n*_ion_ = 50 vs. 9.7% mean RSD, *n*_ion_ = 122 over all portable devices). In the analysis of low molecular weight molecules (e.g., VVOCs and several VOCs), the low number of selective ions with a satisfactory relative abundance is a great challenge for fragment pattern comparisons. Therefore, a threshold of 5% for the relative abundance of *quan* and *identity-confirming (qualifying) ions* (in comparison to 10% in [27, 30]) enabled an extended evaluation in two ranges (*a*_1_ ≤ 25% < *a*_2_).

For the mobile instruments, however, the variance of low-abundance fragments (*a*_1_ < 25%) was clearly higher than that for high-abundance fragments (*a*_2_ ≥ 25%) with a similar ratio of *a*_1_/*a*_2_ means for all identified analytes (~ 1.6 ± 0.1, *n* = 3), while the Stationary had a much lower *a*_1_/*a*_2_ value of ~ 0.9. These differences suggest that, first, the variance with the Stationary is nearly independent of the *relative abundance of the fragments* in each mass spectrum and, second, the poor reproducibility of the mass spectra in portable devices might be most likely related with the higher variance of the low-abundance fragments. Nevertheless, between portable devices, MobH showed considerably better RSD values already approaching the performance of the stationary device for high-abundance fragments.

For MobT, including less volatile compounds to achieve a broader coverage of analytes (MobT DI) considerably lowered the variance compared to MobT HS. We also considered the variance of the mass spectra to be a consequence of the different compounds detected with each of both extraction modes (headspace “HS”-SPME vs. immersion “DI”-SPME); for instance, phenol and chlorinated phenols, characterized by heavier, more abundant fragments and lower RSD values, were only detected by DI-SPME.

As expected, the larger variance in intensity of the chromatographic peaks, first, between extraction modes (e.g., toluene was barely detected through DI, and a broad but less intense peak made the quantification difficult) and, second, among detected compounds (the mean abundance with MobT DI was much lower with a drastic difference in extraction efficiency for 1,2-xylene; see supp. Fig. S.3), did not affect the estimation of the reproducibility of the mass spectra through the height of the peak of selective fragments (as an equivalent to a background-subtracted MS at the apex). Indeed, the % RSD values in MobT DI were better than those in MobT HS.

To identify the reason for the poor precision, the % RSD of all evaluated fragments as a function of specific independent variables was related to (a) the *m/z* (mass) of the fragment and (b) the fragment’s absolute abundance in a linear regression. However, we found no effect of the *mass* on the reproducibility of the fragmentation pattern. (Note, however, that most of the target *m/z* were in a similar range between *m/z* 30 and 100.) From this, we might infer that the difference in the variance of the results for MobT (refer to Table 3) between HS- and DI-SPME seemed to be only related to the abundance of the fragments, but not to the mass of the identified analytes. Though as expected from the already-estimated ratio between mean % RSD values (Table 3), mass spectral variability was found significantly reduced for highly abundant ions for the Stationary and MobE (negative slope, *m*); however, the extent of this influence was rather weak, and we refer to Supp. S.6 for the tabulated results and to Supp. S.7 for illustration and further discussion.

We finally considered that the overall performance of mass spectrometers for *m/z* separation and detection might be dependent on the vacuum level that can be delivered by these systems [3]. When the particle density is increased, collisions with residual gas molecules or space charge effects (electrostatic interaction at larger electron and ion densities in restricted spaces) decrease the *ionization efficiency* and make the *ion transfer* between ion source, analyzer, and detector less successful. At first, in the source, the probability that the analyte interacts with the emitted electron for a successful ionization statistically decreases when the number of other molecules in the ion volume increases, resulting in a lower *ionization efficiency* for the analyte, therefore lower sensitivity (the signal decreases). At the same time, the signal background increases since more residual molecules are ionized, which decreases the sensitivity even further (by raising the noise, the *S/N* ratio will decrease). Second, by the either kinetic (collision) or electrostatic interaction (space charge effects) with residual gas molecules, the ion is deflected from its intended trajectory (directional dispersion) and loses energy (energy/velocity dispersion) [38]. Both lead to a loss of ions during focusing of the ion beam for transfer; also, the signal amplification in the detector will be less efficient at higher pressures due to the lower energy of the ion impacts.

Thus, higher (and less stable) pressures are a very suggestive reason for all observed drawbacks, i.e., low sensitivity, poor reproducibility, and a reduced dynamic range of mobile mass spectrometers [39]. Therefore, we assume that the differences in % RSD might be related to the lower and stable pressure in the Stationary (high vacuum (HV) < 1.0 × 10^−4^ Pa) compared to the mobile systems. To support this assumption, RSD values were also estimated for several compounds of the complex standard mixture for one additional, non-mobile/lab-installed GC–MS quadrupole mass analyzer capable of approaching similar vacuum conditions as the Stationary (for instrumental parameters, refer to Supp. S.2, Table S.2.2).

For instance, the high vacuum (HV) pressure achieved by a *GCMS-QP2010* was 9.9 × 10^−5^ Pa, being slightly lower than the one of the Stationary. As expected, the estimated RSD values (refer to Table 4) in the mass spectra obtained from the GCMS-QP2010 device with the lowest vacuum pressure were better with means of 1.3% (*n*_ion_ = 12) and 2.7% (*n*_ion_ = 12) by using headspace injection with trap or the less sensitive loop mode, respectively, in comparison to the mean of 3.0% (*n*_ion_ = 15) in the Stationary with a slightly higher vacuum pressure.

**Table 4:**
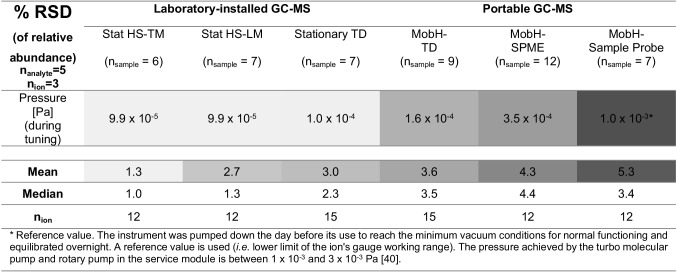
Relative standard deviation (% RSD) of the relative abundance of replicate analysis of three fragments per analyte (exemplarily for five compounds detected in common by most of the instruments: pentafluorobenzene, benzene, toluene, 1,2-xylene, and 3,4-dichlorophenol) for two stationary GC/MS devices with different vacuum pressures: Stat HS-TM (high vacuum (HV) pressure during tuning, 9.9 × 10^−5^ Pa), Stat HS-LM (HV pressure during tuning 9.9 × 10^−5^ Pa), and Stationary (HV pressure during tuning 1.0 × 10^−4^ Pa) in comparison with the three portable systems of the same type of MobH but working under different vacuum pressures and introduction devices: MobH (i.e., TD-GC/MS, vacuum pressure during tuning, 1.6 × 10^−4^ Pa), MobH-SPME (vacuum pressure during tuning, 3.5 × 10^−4^ Pa), and MobH-Sample Probe. (*Note: The latter instrument was pumped down the day before its use to reach the minimum vacuum conditions for normal functioning and equilibrated overnight. Therefore, a reference value (i.e., the lower limit of the ion’s gauge working range) is used for comparison. In general, the pressure achieved by the turbo molecular pump and rotary pump in the service module of MobH is between 1 × 10^−3^ and 3 × 10^−3^ Pa [40]. Trap and loop mode injection are labeled as “Stat HS-TM” and “Stat HS-LM” respectively

Color scheme for % RSD in scale: light gray for favorable and dark gray for less favorable values. Color scheme for increasing pressure from light to dark gray

*n*_*sample*_ number of replicates

Similarly, the % RSD increased with the pressure for three portable GC–MS devices of the same model as MobH (used with different inlets, i.e., TD, SPME, and Probe) but working under different vacuum conditions. In agreement with the ascending vacuum pressure during tuning of these three MobH quadrupole mass analyzers, the estimated mean RSD value also increased, being approximately 3.6% (*n*_ion_ = 15), 4.3% (*n*_ion_ = 12), and 5.3% (*n*_ion_ = 12), respectively, compared to 3.0% (*n*_ion_ = 15) in the Stationary.

On the other hand, though ion trap mass analyzers such as MobT are characterized to achieve optimal performance already at higher pressures [3, 41] eventually making them more suitable for mobile instruments, the higher pressure of MobT might still have led to a poorer than the conventional quadrupole (Stationary) mass spectral reproducibility with MobE and MobH quadrupoles. In agreement, we could not find any significant correlation of the encountered variability, neither as function of the fragments’ absolute abundance in contrast to the quadrupoles (stationary and portable) nor as a function of the mass for this instrument.

Our results suggest as a general tendency that the unfavorable pressure of portable devices compared to that of the *Stationary* causes a poorly reproducible loss of signal response and a higher noise level. However, the differences in the pressure do not seem large enough to justify the observed differences in the variance (i.e., mean 2.4% RSD *n*_ion_ = 39 for 2 stationary devices vs. 4.4% RSD *n*_ion_ = 39 for three portable devices of the same type). Instead, other appearances such as differences in the *pressure stability*, i.e., stronger fluctuations during analysis of replicates, might also cause the observed higher variance.

#### Mobile instruments exhibit a poorer mass spectral similarity for identification with mass spectral libraries

**Fig. 3:**
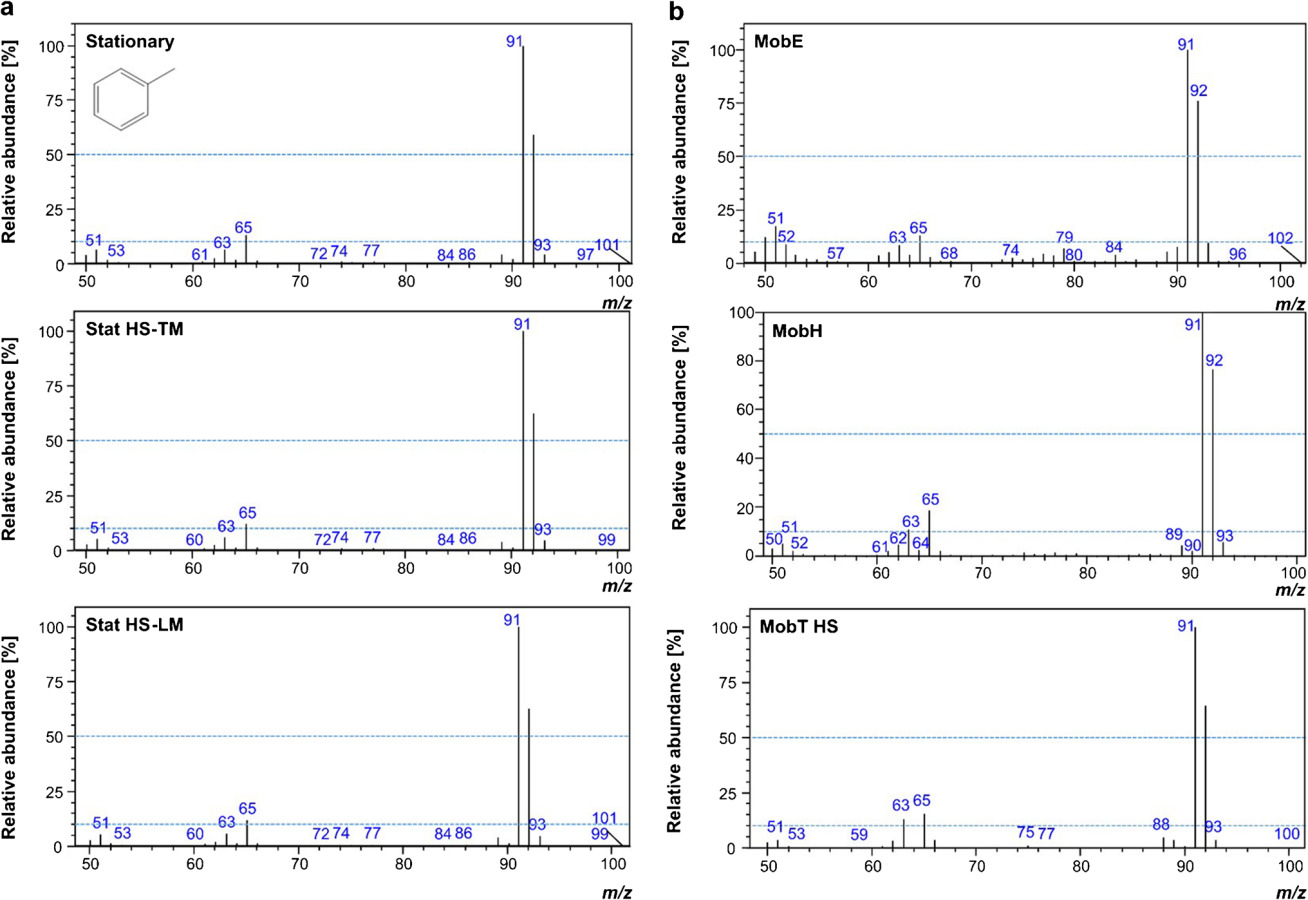
Comparison of the spectra (scan at the apex of the peak) of the aromatic compound, toluene, obtained from **a** stationary GC–MS devices: Stat HS-TM, Stationary HS-LM, and Stationary and **b** portable GC–MS devices: MobH, MobE, and MobT HS, using GCMS solution 4.20 software (Shimadzu, Kyoto, Japan). Trap and loop mode are labeled as “Stat HS-TM” and “Stat HS-LM”, respectively. For MobH, the spectrum was illustrated by ER IQ software version 2.33 (Inficon, Inc., Germany) and formatted. Blue dashed lines have been arbitrarily added to limit 10 and 50% relative abundance and ease the comparison

Having a closer look to the fragmentation patterns obtained with the different devices, we also noticed differences in the mass spectra obtained from the stationary GC–MS systems compared to the ones from portable GC–MS devices, exemplarily illustrated in Fig. 3 (a and b, respectively) for the aromatic compound toluene.

With *m/z* 91 (benzylic cleavage of an H radical from the methyl group) as the base peak, the relative abundance of the molecular radical ion *m/z* 92 for the stationary devices is 61.7 ± 2.6 and 69.5 ± 10.2 for the portable devices. For *m/z* 65, the subsequent neutral cleavage of ethine, the values are 12.4 ± 0.5 for the stationary devices and 16.6 ± 2.1 for the portable devices. Consequently, the abundant radical cleavage is less frequent, and the low-abundance neutral cleavage was somewhat enhanced with the portable devices. This would be a brief example illustrating differences in fragmentation in the ion sources of the mobile instruments vs. the Stationary, as already suggested from the results of our Mann–Whitney *U* tests and illustrated in Supp. S.8 for another compound, phenol, on which larger differences in relative abundance were found for the *m/z* 66, corresponding to the loss of CO from the molecular ion, in MobH (53.1 ± 6.6, *n*_sample_ = 9) and MobT DI (64.0 ± 4.7, *n*_sample_ = 2) than in MobE (30.3 ± 5.3, *n*_sample_ = 3) and compared to the Stationary (41.9 ± 1.5, *n*_sample_ = 7).

Since a distorted fragmentation pattern is expected to have an impact on identification using spectral matching, we further investigated the performance of compound identification with the mobile devices. Identification of unknown compounds without authentic standards is usually accomplished by spectral match with commercial mass spectral libraries. Indeed, tentative identification of compounds has been carried out by comparison with customer or commercial mass spectral libraries and/or literature-based linear retention indexes (LRIs) in many studies using portable devices (even with different mass analyzers, miniature ion trap against quadrupole) [21]. Consequently, it is not only the *reproducibility* that matters here but also the *similarity* to reference spectra from widely accepted databases such as the NIST library (National Institute of Standards and Technology, Gaithersburg, US). The relative abundance normalized to the corresponding NIST reference values is summarized in Table 5.

**Table 5:**
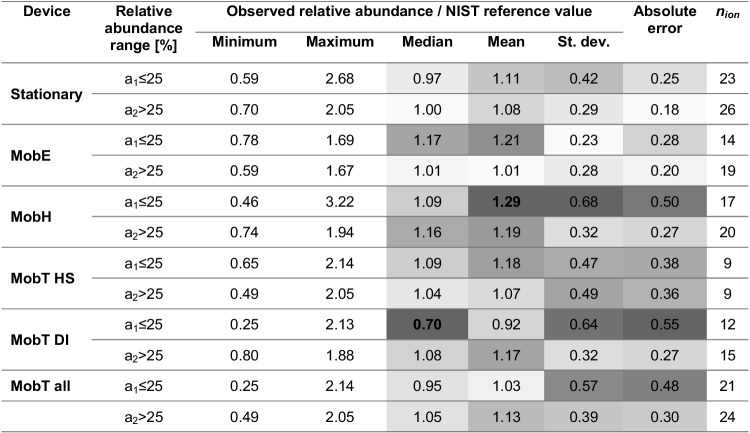
Mass spectral similarity (normalized relative abundance to the reference values of NIST library with an ideal value = (1) colored in scale: light gray for favorable, gray for neutral, and dark gray for unfavorable values per category defined in each column. Three selective mass traces were estimated for the Stationary (*n*_analyte_ = 17, *n*_ion_ = 49, *n*_sample_ = 7, except for propan-2-one *m/z* 44 and butan-2-ol *m/z* 43), MobE (*n*_analyte_ = 13, *n*_ion_ = 33, *n*_sample_ = 3, except for pyridine *m/z* 78, 51, aniline *m/z* 65 and nonane* with filtered base peak), MobH (*n*_analyte_ = 15, *n*_ion_ = 37, *n*_sample_ = 9, only butan-2-ol *m/z* 59, except for nonane with filtered base peak* and pyridine), MobT HS (*n*_analyte_ = 6, *n*_ion_ = 18, *n*_sample_ = 17), and MobT DI (*n*_analyte_ = 9, *n*_ion_ = 27, *n*_sample_ = 2). Note that a few *m/z* with insufficient selectivity were excluded from evaluation. Values are given in two abundance ranges: *a*_1_ ≤ 25% and *a*_2_ > 25%, where *a* is relative abundance

Values in bold represent those with larger differences between the median and mean

*n*_*analyte*_ number of identified analytes, *n*_*ion*_ number of selective ions per relative abundance range, *n*_*samples*_ number of replicates

**Fig. 4:**
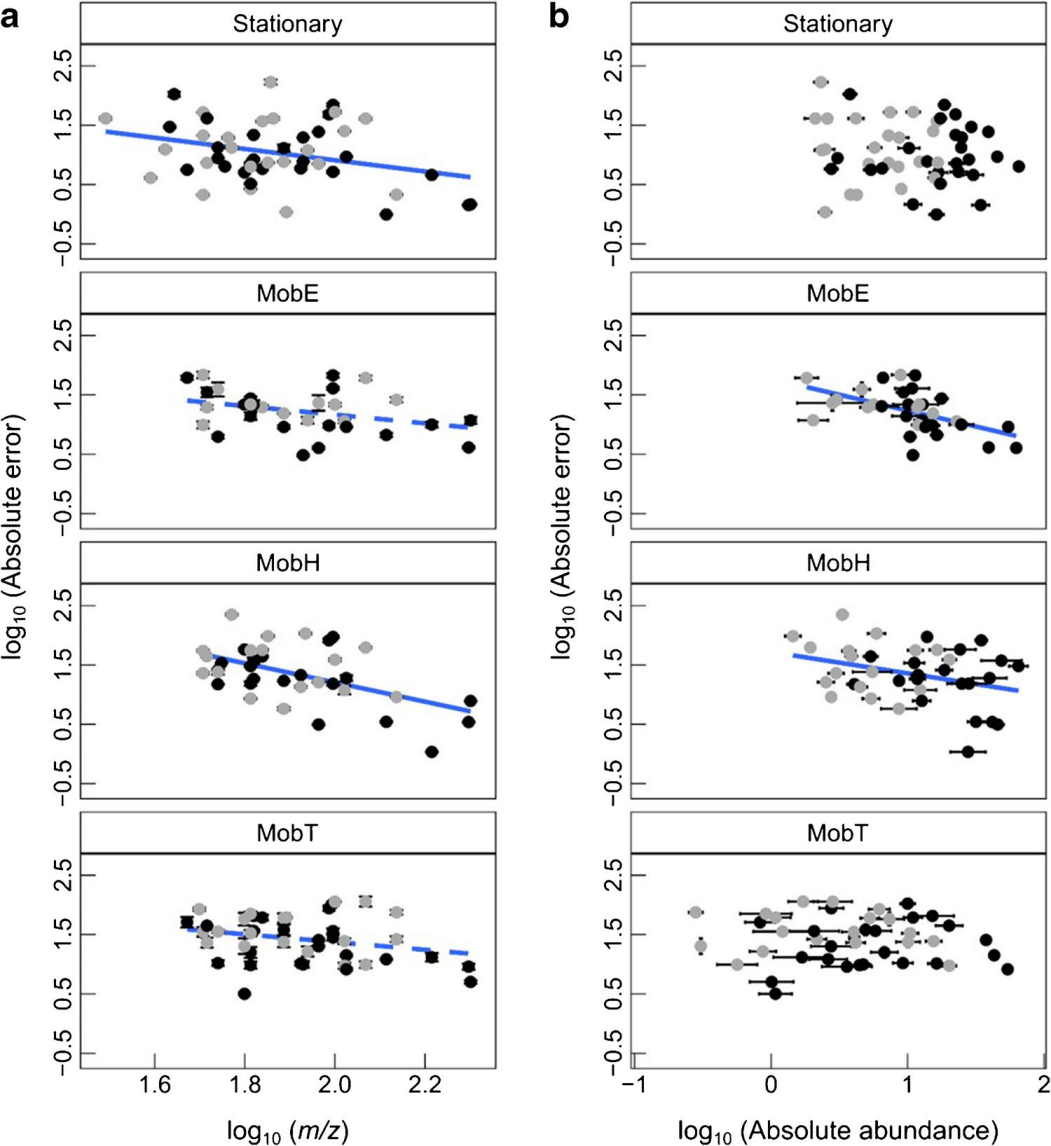
Ggplots representing the mass spectral similarity in terms of % absolute error from each selective ion plotted over (a) their corresponding *m/z* or (b) the fragment’s absolute abundance for the Stationary (*n*_analyte_ = 17, *n*_ion_ = 49, *n*_sample_ = 7, except for propan-2-one *m/z* 44 and butan-2-ol *m/z* 43), MobE (*n*_analyte_ = 13, *n*_ion_ = 33, *n*_sample_ = 3, except for pyridine *m/z* 78 and 51, aniline *m/z* 65, and nonane* with filtered base peak), MobH (*n*_analyte_ = 15, *n*_ion_ = 37, *n*_sample_ = 9, only butan-2-ol *m/z* 59, except for nonane with filtered base peak* and pyridine), MobT HS (*n*_analyte_ = 6, *n*_ion_ = 18, *n*_sample_ = 17), and MobT DI (*n*_analyte_ = 9, *n*_ion_ = 27, *n*_sample_ = 2). Note that a few *m/z* with insufficient selectivity were excluded from evaluation. Terms’ labels: “*n*_analyte_” = number of identified analytes, “*n*_ion_” = number of selective ions from all identified analytes in a particular abundance range and “*n*_sample_” = number of replicates. Dark gray dots illustrate fragments with *a*_1_ ≤ 25% and black dots with *a*_2_ > 25%, where *a* is relative abundance; solid and dashed blue lines represent significant linear correlation (*p* value < 0.05) and marginal linear correlations (0.05 ≤ *p* value ≤ 0.1) upon occurrence, respectively. *Nonane could not be compared with the NIST library since the base peak *m/z* 43 was below the adjusted mass range in MobE and MobH (> *m/z* 45). Tabulated results can be seen in the supplementary section Supp S.6, Table S.6

The highest deviations from the ideal value of 1 of median and mean values (in bold) were found for the low-abundance fragments with MobE, MobH, and MobT, also deviating from normal distribution (median ≠ mean). However, the large standard deviations in general complicate an assessment of the best performance in mass spectral similarity. Considering the lower standard deviation and absolute error, MobE indeed seems to outperform the other portable devices in terms of mass spectral similarity. The consistently higher absolute error of the low-abundance fragments with the mobile devices again suggests poor matching with the NIST reference spectra. We also evaluated the % absolute error of the fragments in dependence on the *mass* (a) and *absolute abundance* (b) of the fragments in a linear regression for the Stationary and mobile systems (Fig. 4).

Significant negative linear correlations were found for all mass spectrometers (*p* value = 0.03, *m* =  − 0.95 and *b* = 2.81 for the Stationary and *p* value < 0.001, *m* =  − 0.62, and *b* = 4.44 for MobH, *p* value = 0.05, *m* =  − 0.73, and *b* = 2.62 for MobE, and *p* value = 0.08, *m* =  − 0.65, and *b* = 2.68 for MobT) as a function of the *mass* (i.e., mass spectral similarity improves for heavier fragments). The fragments’ absolute abundance was negatively correlated only for the two portable quadrupole analyzers. Although the effect seems low judging from the slope and overall appearance of the graphs, spectral matching might benefit from restricting the searched *m/z* range to the lower end and introducing an intensity threshold; the same would be true for the selection of a *quan* ion.

Variations in the fragmentation pattern were already reported in a previous study [42] with a portable GC–MS Tridion™-9 (Torion Technologies Inc.). Such differences may be related to the different internal energy of the ions which in turn again may be a consequence of the pressure in the ion source. Moreover with respect to MobT ion trap spectra were often reported to suffer from poor spectral matches and a lower comparability across different instruments in comparison with EI reference spectra from beam-type analyzers normally used for creating the NIST library entries [37]; in trap analyzers, the deceleration of ions to less internal energy may result in a partially different fragment pattern (e.g., [43]). However, since our values do not suggest a worse performance of the portable trap instrument in comparison to the quadrupole analyzers, we infer that the improved results for spectral matching might also be efficiently achieved improving the mass spectral *reproducibility* over the entire evaluated mass range.

#### Mobile instruments exhibit a lower sensitivity

**Fig. 5:**
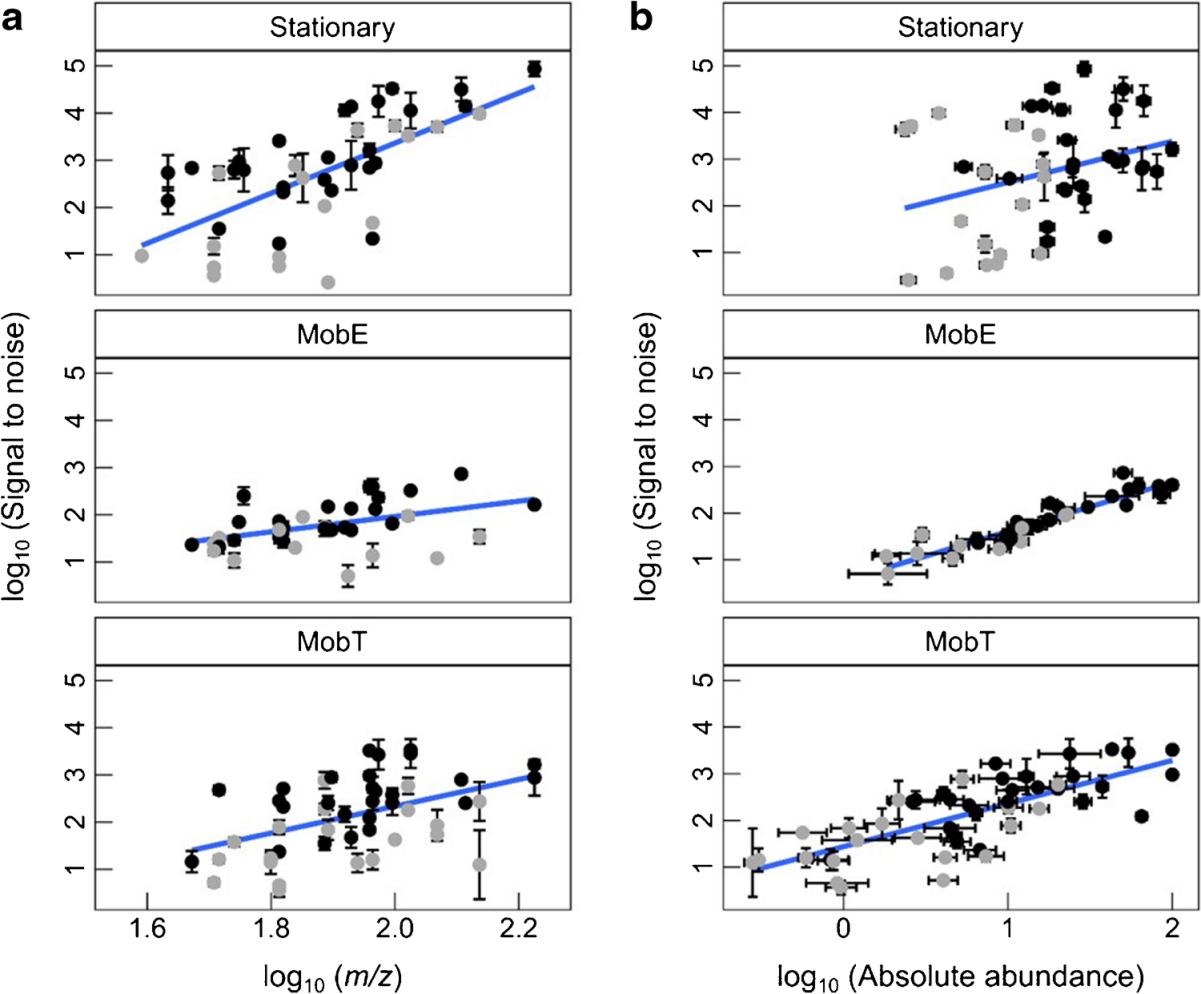
Ggplots representing the *S/N* (RMS calculation) of four selective mass traces per compounds in logarithmic scale in the Stationary and mobile instruments. *S/N* values are plotted over **a** *m/z* and **b** fragment’s absolute abundance. Representative analytes were used for illustration in the Stationary (*n*_analyte_ = 11, *n*_ion_ = 44, *n*_sample_ = 7), MobE (*n*_analyte_ = 11, *n*_ion_ = 36, *n*_replicate_ = 3, except for chloroform *m/z* 87, pentafluorobenzene *m/z* 168, pyridine *m/z* 51 and 78, nonane *m/z* 128, aniline *m/z* 65, 4-chlorophenol *m/z* 130 and 100) and MobT HS (*n*_analyte_ = 5, *n*_ion_ = 20, *n*_sample_ = 17), and MobT DI (*n*_analyte_ = 9, *n*_ion_ = 27, *n*_sample_ = 2). Note that a few *m/z* with insufficient selectivity were excluded from evaluation of MobE. Dark gray dots illustrate fragments with *a*_1_ ≤ 25% and black dots with *a*_2_ > 25%, where *a* is relative abundance; blue lines represent significant linear correlations upon occurrence. Terms’ labels: “*n*_analyte_” = number of identified analytes, “*n*_ion_” = number of selective ions per particular abundance range, and “*n*_sample_” = number of replicates. (Note: particularly for MobE, *S/N* could not be calculated through RMS for very low abundantc ions by the applied software.) Tabulated results of the statistical analysis are given in the supplementary section (Supp. S.6, Table S.6)

For comparing the sensitivity of the instruments, we used the log-transformed signal-to-noise ratio (RMS) plotted over (a) the fragment mass and (b) the fragment’s absolute abundance of four selective mass traces per analyte (Fig. 5).

At a first glance, the Stationary again clearly outperforms the mobile instruments exhibiting the highest *S/N* values. Indeed, the median *S/N* in the Stationary compared to the MobE was about 8 times higher for 11 analytes covering a wide range of volatility and chemical classes (for tabulated values, refer to Supp. S.9). Significant positive correlations of the *S/N* and the mass of the fragment were found for all instruments (Stationary *p* < 0.001, *m* = 5.31, and *b* =  − 7.25; MobE *p* < 0.01, *m* = 1.62, and *b* =  − 1.28; and MobT *p* < 0.001, *m* = 2.84, and *b* =  − 3.35) with the Stationary most influenced (larger slope, higher degree of significance). Very likely, the lower noise for higher *m/z* is responsible for the improved *S/N*.

At constant noise, *S/N* should linearly increase with the abundance for all evaluated instruments. Exemplarily, for benzene, the molecular ion *m/z* 78 (base peak, relative abundance 100%) and the fragment *m/z* 77 (observed relative abundance of 26.4 ± 2.1% for MobE and 24.7 ± 3.2% for Stationary) had an estimated *S/N* of 149 and 53 in the MobE, and 1151 and 389 in the Stationary, respectively. Indeed, significant linear correlations between *S/N* and the fragment’s absolute abundance were obtained for the Stationary (*p* value = 0.04, *m* = 0.88, and *b* = 1.62) and for both portable devices, MobE (*p* value < 0.001, *m* = 1.06, and *b* = 0.55) and MobT (*p* < 0.001, *m* = 0.92, and *b* = 1.44) (see also Supp S.6, Table S.6). Both portable devices, independent of the mass analyzer and introduction device, showed a shorter *y* axis scale (small range of *S/N* values) but larger *x* axis range (larger range of absolute abundance), i.e., a lower fragment selectivity. In conclusion, the fact that the *S/N* along the fragments’ absolute abundance (Fig. 5b) increased to a different extent on the different devices suggests that instruments with a shorter *y* range (*S/N* values) and larger *x* range (abundance) struggle with higher noise values counteracting the positive effect of signal increase. As discussed for the poorer reproducibility, we suggest that this loss of sensitivity might also be related to a higher pressure with the mobile instruments, mostly affecting the low-abundance fragments.

In agreement with our findings, other studies also report on poorer sensitivity and detection limits of portable devices compared to benchtop GC–MS instruments. For example, scent compounds could not be quantified with the sample probe of the Hapsite Smart Plus ER (Inficon) at concentrations met under field conditions (10 times the background noise) and the poor precision of such measurements also complicates the quantitative analysis [19]. In a study of da Silva Pinheiro et al. [15], analysis of 2,6-dimethylphenol with portable TD/GC–MS resulted in a detection limit higher by a factor of 3.

*S/N* was not related to the analytes’ boiling point except in MobT HS (refer to Supp S.10). The results for MobT HS suggest that this effect might be related to the still pending optimization of the sample introduction inlet since the results were considerably improved, for instance, using a higher temperature during compound desorption, or using direct immersion instead of extraction from the headspace. This confirms the still urgent need to improve the transfer system of mobile instruments for successful multicomponent analysis, especially for VVOCs (as already described in “Signal response patterns of the VOC standard mixture after TD/GC–MS analysis differ between the evaluated instruments”).

## Conclusions

In comparison to the performance of stationary lab equipment, mobile GC–MS systems still show shortcomings in their performance and limitations in quantification of complex volatile profiles. Different signal response patterns, a lower signal-to-noise ratio, lower mass spectral reproducibility, and similarity observed with these instruments indicate the need for development of mobile devices to achieve: (i) a wider coverage of analytes, (ii) a more sensitive detection, and (iii) a reliable identification of compounds.

The mobile systems had a *poorer mass spectral reproducibility* which improved with the relative abundance of the fragments while the conventional system exhibited an almost similar variance of low- as well as high-abundance ions (relative abundance) over the investigated mass range. To improve the precision of quantification using mobile quadrupole instruments, we suggest to use selective ions with a general threshold of 25% relative fragment abundance for quantification, particularly for *m/z* < 100; smaller fragments 45 < *m/z* < 100 can still be used for precise determinations but should preferably meet this intensity threshold (refer to Supp. S.11).

In addition, we found the *mass spectra* from mobile instruments *less similar* in the search against standard reference libraries. In more detail, mass spectral similarity with these instruments was *worse* for *lighter and low-abundant fragments*, so that fragments < *m/z* 100 and< 25% intensity may be disregarded respectively less considered to improve spectral hits for mass spectral search (refer to Supp. S.12). We further suggest to consider the assessment of mass spectral similarity respectively comparability in performance tests of mobile devices.

The *S/N* increased with increasing *m/z* independent of the signal abundance (illustrating the benefit of the lower noise with increasing mass range) to a different extent in the different instruments, i.e., less in both, the miniaturized quadrupole mass analyzer MobE compared with the miniaturized ion trap MobT.

Thus, taking together the poorer sensitivity, reproducibility, and comparability across instruments [3], we conclude that the still limited performance in comparison with conventional devices is the consequence of an instrumental configuration to save the limited resources for mobile devices upon miniaturization for mobilization of mass spectrometers (space requirements and media and energy consumption). We suggest that analysis with mobile instruments will particularly benefit from lowering the pressure in the ion source and analyzer region. Apart from a higher pump capacity, this goal might be also achieved further decreasing the dimensions of the ion source/analyzer region. Although we did not observe a generally better performance of the trap analyzer usually tolerating higher pressure, the variance in analysis of the detected analytes might be carefully considered when this kind of analyzer is alternatively implemented to mobile mass spectrometers. Moreover, for these mass spectrometers, the partially different EI fragment patterns need to be also taken in consideration when anticipating compound identification by traditional mass spectral databases, which usually contain reference spectra obtained from instruments with beam-type analyzers (quadrupoles and sector fields). Technological advantages leading to (i) optimized sample inlet systems for VVOCs, (ii) a lower pressure in the ion source/analyzer region, and/or (iii) the development and implementation of analyzers working at higher pressures are anticipated to provide the highest improvements in mobile GC–MS.

## Supplementary Information

Below is the link to the electronic supplementary material.

Supplementary file1 (PDF 1.38 MB)
