# Supplementary material for: A comparison between mobile and stationary gas chromatography–mass spectrometry devices for analysis of complex volatile profiles
Source: Anal Bioanal Chem. 2022 Nov 17;415(1):137–55. doi: 10.1007/s00216-022-04391-y (PMC9672629; doi:10.1007/s00216-022-04391-y)
Supplement: Supplementary file 1 — Supplementary file1 (PDF 1.38 MB) [file 216_2022_4391_MOESM1_ESM.pdf]

Appendix. Electronic supplementary material

A comparison between mobile and stationary gas chromatography – mass spectrometry devices for analysis of complex volatile profiles

Andrea Marcillo[1,a,*], Juan C. Baca Cabrera[2,b], Anja Widdig[3,4,5], Claudia Birkemeyer[1,*]

[1] Research Group of Mass Spectrometry, Institute of Analytical Chemistry, Faculty of Chemistry and Mineralogy, University of Leipzig, Linnéstr. 3, 04103 Leipzig, Germany.

[2] Grassland Group, Technical University of Munich, Alte Akademie 12, 85354, Freising, Germany.

[3] Research Group of Behavioral Ecology, Institute of Biology, Faculty of Life Sciences, University of Leipzig, Talstr. 33, 04103 Leipzig, Germany.

[4] Research Group of Primate Behavioural Ecology, Department of Human Behaviour, Ecology and Culture, Max-Planck Institute for Evolutionary Anthropology, Deutscher Platz 6, 04103 Leipzig, Germany.

[5] German Center for Integrative Biodiversity Research (iDiv), Deutscher Platz 5E, 04103 Leipzig, Germany.

now at

[a] Institute of Energy and Climate Research (IEK-8), Forschungszentrum Jülich GmbH, Wilhelm-Johnen-Strasse, 52428 Jülich, Germany.

[b] Institute of Bio- and Geoscience, Agrosphere (IBG-3), Forschungszentrum Jülich GmbH, Wilhelm-Johnen-Strasse, 52428 Jülich, Germany.

* Corresponding author

Correspondence to:

Dr. Claudia Birkemeyer

University of Leipzig, Faculty of Chemistry and Mineralogy

Institute of Analytical Chemistry, Research Group of Mass Spectrometry

Linnéstr. 3, 04103 Leipzig, Germany

Tel.: +49-341-9736092

e-mail: birkemeyer@chemie.uni-leipzig.de

Andrea Marcillo
Forschungszentrum Jülich GmbH
Institute of Energy and Climate Research (IEK-8)
Wilhelm-Johnen-Strasse, 52428 Jülich, Germany
e-mail: a.marcillo.lara@fz-juelich.de

S.1 Instrumental parameters of TD/GC-MS analysis

Table S.1 Experimental parameters for sample preparation, introduction devices and analysis of complex volatile mixtures by different mobile GC-MS systems compared to a stationary device.

Phase	Category	Parameter	Stationary	MobE	MobH	MobT
**1. Sample preparation**	**1.1 Conditioning**	*Instrument*	TD Clean Cube unit flushed with nitrogen at 50 mL/min from a generator	In-house adapted GC oven as a unit for conditioning of larger TD tubes and flushed with nitrogen at 100 mL/min from a generator	TD Clean Cube unit flushed with nitrogen at 50 mL/min from a generator	N/A
		Temperature program	40 to 250 °C, 152 min	40 to 200 °C, 150 min	40 to 250 °C, 152 min	N/A
	**1.2 Spiking**[a]	*Sampler type*	TD tubes: 6.35 mm O.D. x 89 mm length, Tenax TA 60/80 (Supelco/Sigma-Aldrich, Taufkirchen, Germany)	TD tubes: 8 mm O.D. x 110 mm length, Tenax TA 60/80 (Günther Karl OHG, Gau-Algesheim, Germany)	TD tubes: 6.35 mm O.D. x 89 mm length, Tenax TA 60/80 (Supelco/Sigma-Aldrich, Taufkirchen, Germany)	SPME syringe with pre-conditioned 65 µm PDMS-DVB fibers (PerkinElmer, Germany)
		Injected volume	1 µL standard mix	1 µL standard mix	1 µL standard mix	Head-space (HS) or direct immersion (DI) from / in 0.4 mL standard mix dissolved in methanol in a 9.1 mL total volume vial
		Collection time	13 min	13 min	13 min	0.5 min extraction time
		Inert gas, flow rate	Nitrogen at 100 mL/min	Nitrogen at 100 mL/min	Nitrogen at 100 mL/min	N/A
		Concentration range	0.8 - 1.0 mM	0.8 - 1.1 mM	0.8 - 1.0 mM	0.8 - 1.0 mM
		Replicates	*n = 7*	*n = 3*[b]	*n = 9*	*n = 17 (HS); n = 2 (DI)*
**2. TD/GC-MS analysis**	**2.1 Thermal desorption and interface**	*Flow rate*	Purge at 60 mL/min			
		Temperature, ramp rate	Valve 250 °C, trap cool −19 °C, trap heat 250 °C, interface 260 °C, transfer line 260 °C and block 250 °C	Inlet valve 230 °C, desorption 240 °C	Initial 40 °C, final 290 °C, 1.5 °C/s. Membrane 120 °C, valve oven 110 °C, heated lines 110 °C	Injector 270 °C and transfer line 250 °C
		Time	Cycle: 14 min (8 min sampling, 6 min desorption, 3 min waiting)	Hold 120 s, loading 60 s	Cycle time 7 min	Desorption time 5 s
		Concentrator	Tenax TA	N/A	Tribed	N/A
		Injection mode	Split ratio 10	Splitless (60 s)	Splitless	-

**2.2 Gas chromatograph**	*Mobile phase*	Helium 5.0 (alphagaz Air Liquide, Düsseldorf, Germany) at a column flow of 1.55 mL/min and linear velocity of 35 cm/s	Ambient air prefiltered by a carbon filter (Bruker Daltonic GmbH, Leipzig, Germany) at a capillary flow of 1.5 mL/min and pressure of 700 mbar	VOC-Free Nitrogen with total hydrocarbons < 0.5 ppm and argon < 40 ppm (Scott Specialty Gases, Inficon, Germany)	Helium (≥ 99.9%)
	Stationary phase	Two GC columns connected with each other: Rxi-1 ms, 30 m × 0.25 mm ID, 0.25 µm df, and SGE Analytical Science BPX50, 2 m × 0.15 mm ID, 0.15 µm df (Restek GmbH, Bad Homburg vor der Höhe, Germany)	High temperature GC column: DB1, 15 m x 0.32 mm ID, 5.00 µm df (Bruker Daltonic GmbH, Leipzig, Germany)	GC column: dimethyl polysiloxane phase 100%, 15 m x 0.25 mm ID x 1.0 µm df (Inficon, Germany)	GC column: MXT5, 5 m x 0.1 mm ID x 0.4 µm df (PerkinElmer, Germany)
	Phase composition	Rxi-1ms: 100% dimethyl polysiloxane<br>BPX50: 100% methylphenyl polysiloxane	100% dimethyl polysiloxane	100% dimethyl polysiloxane	95% dimethyl / 5% diphenyl polysiloxane
	Temperature program	Initial temperature 50 °C for 0.5 min, increased by 10 °C/min to 250 °C for 19.5 min, and kept for another 5 min	Initial temperature 50 °C for 1.5 min, increased by 5 K/min to 120 °C during 14 min, then increased by 10 K/min to 240 °C for 12 min, kept for another 3 min	Initial temperature 60 °C for 1 min, increased by 6 °C/min to 80 °C for 3.3 min, then by 12 °C/min to 120 °C for 3.3 min, then by 26 °C/min to 200 °C for 3.1 min, kept for 3.3 min	Initial temperature 50 °C for 10 s, increased by 2 °C/s to 270 °C for 110 s, kept for another 60 s
**2.3 Mass spectrometer**	*Ionization source*	Electron impact (EI) at 70 eV, 200 °C	Electron impact (EI) at 70 eV, 150 °C	Electron impact (EI) at 70 eV	Electron impact (EI) at 70 eV
	Mass analyzer	Quadrupole<br>Mass range: *m/z* 30-300	Quadrupole<br>Mass range: *m/z* 45-400	Quadrupole<br>Mass range: *m/z* 45-300	Toroidal ion trap<br>Mass range: *m/z* 43-500

[a]The period between the preparation of the standard solution and the spiking of thermal desorption tubes was less than 24 hours. Before measurements with SPME, the standard mixture was stored and kept at < 4 °C.
[b]Replicates used for evaluation at different concentration ranges: ~0.3 mM (*n = 2*), 0.8 - 1.1 mM (*n = 3*), ~2 mM (*n = 1*).

S.2 Variance of replicate analyses for systems working at different vacuum pressures

Relative standard deviation (% RSD) of the relative abundance of replicate analysis of three fragments per analyte was already shown and compared between the Stationary and the portable devices, MobE, MobH and MobT (refer to "Mobile instruments exhibit a poorer mass spectral reproducibility" in the manuscript). Considering that the overall performance of the miniaturized mass spectrometers with mass analyzers operating under low pressure (*e.g.* quadrupoles) is highly influenced by the vacuum system [1], we suggest that the encountered poorer reproducibility might be related to poorer and/or less stable vacuum conditions. To find further evidence for this assumption, % RSD values were also estimated for several compounds of the complex standard mixture (5 analytes) analyzed with an additional stationary GC-MS device capable of achieving better vacuum pressures. We searched for the differences on the reproducibility of the EI mass spectra of stationary GC-MS devices to determine their potential correlation with the vacuum pressure (achieved during tuning) in comparison with the reproducibility of three portable devices of the same type as MobH with different sample introduction devices and working under poorer vacuum conditions. For this additional test, the experimental parameters are described in the following Table S.2.1.

Table S.2.1 Experimental parameters for sample preparation, introduction devices and analysis of several VOCs by different stationary and portable GC-MS systems. Additional devices working under different vacuum pressures for evaluation of mass spectral reproducibility. "n_{sample}" = number of replicates.

Parameter	Stat HS-TM	Stat HS-LM	MobH-SPME	MobH-Sample Probe
**1. Sample preparation**				
Injected volume	Head-space (HS) in 1 µL standard mix dissolved in methanol added to 1 mL water (HiPerSolv Chromanorv, VWR Chemicals, France) in a 10 mL total volume vial	Head-space (HS) in 1 µL standard mix dissolved in methanol added to 1 mL water (HiPerSolv Chromanorv, VWR Chemicals, France) in a 10 mL total volume vial	SPME syringe with pre-conditioned 75 µm PDMS-DVB fibers (Inficon, Germany) exposed 15-30 s to the head-space (HS) from / in 100 µL standard mix dissolved in methanol in a 1.5 mL total volume vial	Sample probe exposed 30 s to the head-space (HS) from / in 100 µL standard mix dissolved in methanol in a 1.5 mL total volume vial
Concentration	~ 1.0 mM	~ 1.0 mM	0.8 - 1.0 mM	0.8 - 1.0 mM
Replicates	(n_{sample} = 6)	(n_{sample} = 7)	(n_{sample} = 12)	(n_{sample} = 7)
**2. GC-MS analysis**				
**2.1 Introduction device**				
Mode	Head-space, trap mode	Head-space, loop mode	SPME	Sample probe
Temperatures	Oven 60 °C, sample line 150 °C, transfer line 160 °C, trap desorption 300 °C	Oven 60 °C, sample line 150 °C, transfer line 160 °C	Oven 60 °C, membrane 120 °C, valve oven 110 °C, heated lines 110 °C and SPME 260 °C	Oven 60 °C, membrane 120 °C, valve oven 110 °C, heated lines 110 °C and probe 40 °C
Time	Equilibrating 10 min, pressurizing 0.5 min, load 2.0 min, and injection 1 min	Equilibrating 5 min, pressurizing 1 min, load 0.5 min, and injection 1 min	SPME line purge 1 min, concentration 3 min, desorption 30 s	Line purge 1 min, concentration 1 min, desorption 30 s
Concentrator	Tenax TA	N/A	Tribed	
**2.2 Gas chromatograph**				
Injection mode	Split ratio 10	Split ratio 5	Splitless	Splitless
Mobile phase	Helium 5.0 (alphagaz Air Liquide, Düsseldorf, Germany) at a column flow of 0.95 mL/min and linear velocity of 35.1 cm/s	Helium 5.0 (alphagaz Air Liquide, Düsseldorf, Germany) at a column flow of 0.95 mL/min and linear velocity of 35.1 cm/s	VOC-Free Nitrogen with total hydrocarbons < 0.5 ppm and argon < 40 ppm (Scott Specialty Gases, Inficon, Germany)	
Stationary phase	Rxi-5Sil MS, 30 m x 0.25 mm ID, 0.25 µm df (Restek Corp., Pennsylvania, USA)	Rxi-5Sil MS, 30 m x 0.25 mm ID, 0.25 µm df (Restek Corp., Pennsylvania, USA)	Dimethyl polysiloxane phase 100%, 15 m x 0.25 mm ID x 1.0 µm df (Inficon, Germany)	
Temperature program	Initial temperature 35 °C for 2 min, increased by 5 °C/min to 100 °C for 5 min, and finally increased by 25 °C/min to 240 °C for 15 min	Initial temperature 35 °C for 1 min, increased by 10 °C/min to 250 °C for 21.5 min, and kept for another 5 min	Initial temperature 60 °C for 1 min, increased by 6 °C/min to 80 °C for 3.3 min, then by 12 °C/min to 120 °C for 3.3 min, then by 26 °C/min to 200 °C for 3.1 min, kept for 3.3 min	
**2.3 Mass spectrometer**				
Ionization source	Electron impact (EI) at 70 eV	Electron impact (EI) at 70 eV	Electron impact (EI) at 70 eV	
Mass analyzer	Quadrupole	Quadrupole	Quadrupole	
	Mass range: *m/z* 29-500	Mass range: *m/z* 29-500	Mass range: *m/z* 41-300	Mass range: *m/z* 45-300

As mentioned in “Experimental design and instrumental parameters of analysis on mobile TD/GC-MS instruments” in the main manuscript, a *GCMS-QP2010* system composed of a gas chromatograph *GC-2010 Plus* coupled to a quadrupole mass spectrometer (Shimadzu, Kyoto, Japan) was used, and the samples were introduced into the instrument by a head space system *HS-20* (Shimadzu, Kyoto, Japan) using both, trap and loop mode and labelled as “Stat HS-TM” and “Stat HS-LM”, respectively. The high vacuum (HV) pressure of the instrument reached a value of 9.9 x 10^{-5} Pa, being slightly lower than the HV pressure of the already evaluated Stationary (1.0 x 10^{-4} Pa). In Table S.2.2, we present a comparison of the estimated RSD values among instruments together with the vacuum pressure obtained at the corresponding tuning or a reference value, when this data was not available. Similar to the other instruments, the evaluation of the data was performed using the *GCMS solution* software version 4.20 as already described in “Data evaluation” in the manuscript.

Table S.2.2 Relative standard deviation (% RSD) of the relative abundance of replicate analysis of three fragments per analyte (exemplarily for 5 compounds detected in common by most of the instruments) for two stationary GC/MS devices with different vacuum pressures: Stat HS-TM (high vacuum (HV) pressure during tuning, 9.9 x 10^{-5} Pa), Stat HS-LM (HV pressure during tuning 9.9 x 10^{-5} Pa) and Stationary (HV pressure during tuning 1.0 x 10^{-4} Pa) in comparison with the three portable systems of the same type of MobH but working under different vacuum pressures and introduction devices: MobH (vacuum pressure during tuning, 1.6 x 10^{-4} Pa), MobH-SPME(vacuum pressure during tuning, 3.5 x 10^{-4} Pa) and MobH-Sample Probe. (*Note: This latter instrument was pumped down the day before its use to reach the minimum vacuum conditions for normal functioning and equilibrated overnight. Therefore, a reference value (*i.e.* the lower limit of the ion's gauge working range) is used for comparison. The pressure achieved by the turbo molecular pump and rotary pump in the service module is between 1 x 10^{-3} and 3 x 10^{-3} Pa [2].) Color scheme for % RSD in scale: light grey for favorable and dark grey for less favorable values. Color scheme for increasing pressure from light to dark grey. Terms’ labels: “n_{sample}” = number of replicates, “n.d.” = not detected.

**% RSD (Relative abundance)**			**Laboratory-installed GC-MS**			**Portable GC-MS**		
			Stat HS-TM (n_{sample} = 6)	Stat HS-LM (n_{sample} = 7)	Stationary (n_{sample} = 7)	MobH (n_{sample} = 9)	MobH-SPME (n_{sample} = 12)	MobH-Sample Probe (n_{sample} = 7)
**No.**	**Analyte**	**Pressure [Pa]**	9.9×10^{-5}	9.9×10^{-5}	1.0×10^{-4}	1.6×10^{-4}	3.5×10^{-4}	Reference* 1.0×10^{-3}
		m/z						
		99	2.2	9.3	6.4	3.8	3.8	2.5
**6**	1,2,3,4,5-Pentafluorobenzene	137	2.2	5.3	2.3	3.1	4.2	1.9
		117	2.5	7.9	3.9	1.7	3.5	2.5
		77	0.7	1.2	9.6	0.9	1.8	1.4
**7**	Benzene	51	0.7	0.7	1.1	3.9	3.2	17.7
		52	0.9	0.8	2.0	3.2	5.0	15.3
		92	0.7	0.9	0.6	2.0	3.3	1.0
**10**	Toluene	65	0.9	1.3	1.4	3.5	5.8	5.6
		51	1.0	1.6	2.7	4.8	6.3	4.2
		106	1.3	1.0	1.7	5.2	5.6	5.3
**12**	1,2-Xylene	77	1.1	1.5	1.5	5.6	5.1	1.4
		105	1.2	1.3	2.3	3.5	4.5	5.2
		164	n.d.	n.d.	1.3	1.6	n.d.	n.d.
**18**	3,4-Dichlorophenol[e]	99	n.d.	n.d.	4.7	6.3	n.d.	n.d.
		63	n.d.	n.d.	3.8	5.1	n.d.	n.d.
		**Mean**	**1.3**	**2.7**	**3.0**	**3.6**	**4.3**	**5.3**
		**Median**	1.0	1.3	2.3	3.5	4.4	3.4
		**n_{ion}**	12	12	15	15	12	12

* The instrument was pumped down the day before its use to reach the minimum vacuum conditions for normal functioning and equilibrated overnight. A reference value is used (*i.e.* lower limit of the ion's gauge working range). The pressure achieved by the turbo molecular pump and rotary pump in the service module is between 1×10^{-3} and 3×10^{-3} Pa [2].

As expected for permanently installed GC-MS devices, such as Stat HS-TM and Stat HS-LM with the lowest vacuum pressure, the estimated RSD values (refer to Table S.2.2) were lower with means of 1.3% (n_{ion} = 12) and 2.7% (n_{ion} = 12), respectively in comparison to the 3.0% (n_{ion} = 15) with the Stationary featuring a higher pressure.

On the other hand, for the three portable GC-MS devices different by introduction device and working at higher pressures, higher RSD values were found. In general, for MobH, the vacuum pressure is approximately 10 times higher than the Stationary by considering only the pressure achieved by the turbo molecular pump and the rotary pump in the service module (between 1 x 10^{-3} and 3 x 10^{-3} Pa [2]), but subsequently improved most likely by the effect of a non-evaporative getter (NEG) pump and a smaller sputter-ion pump included in the analytical unit. Own values present a HV pressure of 1.6 x 10^{-4} Pa for MobH with TD tubes as introduction device and 3.5 x 10^{-4} Pa for MobH with SPME. The third portable instrument with a sample probe inlet was pumped down the day before its use to reach the minimum vacuum conditions for normal functioning and was equilibrated overnight. In this case, therefore, a reference value (*i.e.* the lower limit of the ion's gauge working range, 1 x 10^{-3} Pa) was used here for comparison. In agreement with the ascending vacuum pressure during tuning of these three different MobH systems with variable introduction devices (*i.e.* TD, SPME and sample probe) and quadrupole mass analyzers, the means of the estimated RSD values also increased, being approximately 3.6% (n_{ion} = 15), 4.3% (n_{ion} = 12) and 5.3% (n_{ion} = 12), respectively, compared to 3.0% (n_{ion} = 15) in the Stationary.

In sum, we found that the mean of RSD values for two different stationary devices, working under three different set up configurations, was lower (2.4%, n_{ion} = 39) than in the three portable devices of the same type and different vacuum pressures (4.4%, n_{ion} = 39).

S.3 Comparison of signal response profiles for a complex standard VOC mixture between stationary and portable TD/GC-MS devices and a mobile SPME/GC-MS system

Fig. S.3 presents the comparison of signal response profiles of the standard mixture of 18 compounds analyzed on a conventional TD/GC-MS (Stationary) and two TD/GC-MS mobile devices (MobE and MobH). In addition, the results from a SPME/GC-MS portable system (MobT) were also added here for comparison under two different extraction modes: head-space (HS) and direct immersion (DI).

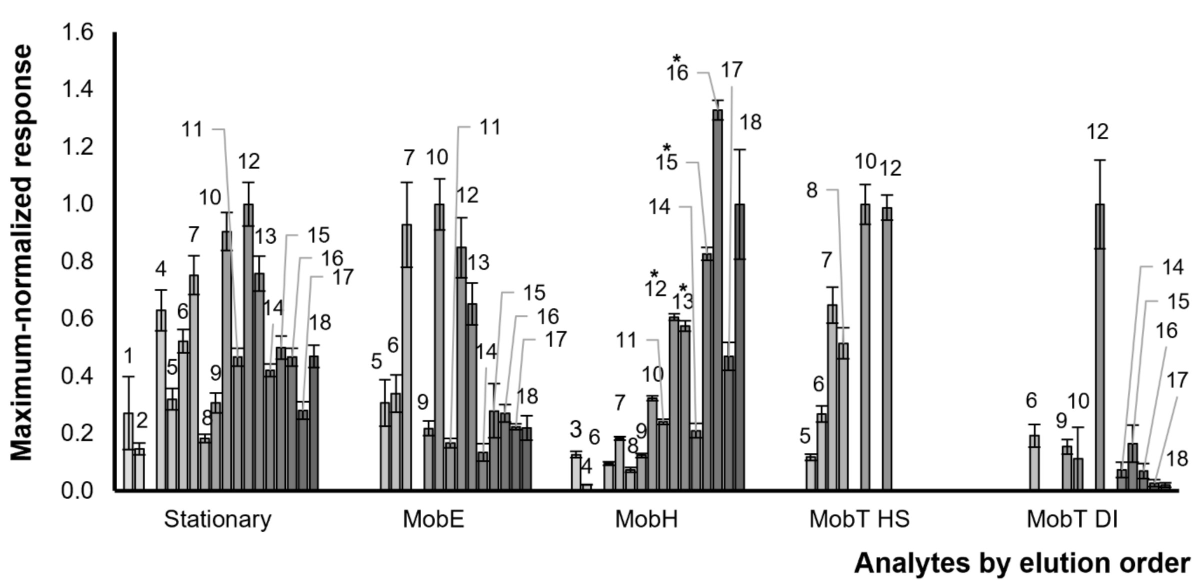

Fig. S.3 Maximum-normalized response of the standard mixture of 18 compounds (for identification of labelled numbers and further detail on detected compounds, refer to Table 1 and Supp. A.4) analyzed on a TD/GC-MS stationary and two TD/GC-MS mobile devices, MobE and MobH. In addition, a SPME/GC-MS portable system (MobT) was also added here for comparison. Standards were dissolved in methanol and spiked in Tenax TA thermal desorption tubes (1 µL sample volume, 0.8 - 1.1 mM concentration range) followed by 13 min collection time using nitrogen passing through the sorbent bed at 100 mL/min flow rate. A SPME syringe with a PDMS/DVB fiber was used for sampling the head-space of a 0.4 mL complex standard mixture dissolved in methanol in a closed chamber (9.1 mL total volume glass vial) during 30 s extraction time followed by 5 s desorption time and splitless as injection mode (labelled as MobT HS). In a second approach, the fiber was directly immersed in the standard solution during 30 s extraction time followed by 5 s desorption time and splitless as injection mode (labelled as MobT DI). The integrated areas (each replicate) from the *quan ion* (m/z_1) of all identified analytes in the standard mixture were normalized to the mean area of the *quan ion* with the maximum value among all compounds (labeled as "*maximum-normalized response*"). 1,2-Xylene (12), toluene (10), 3,4-dichlorophenol (18), toluene (10), and 1,2-xylene (12) were used for maximum normalization for the Stationary, MobE, MobH, MobT HS and MobT DI devices, respectively. The resulting identified compounds in the Stationary ($n_{analyte}$ = 17, n_{sample} = 7), MobE ($n_{analyte}$ = 13, n_{sample} = 3), MobH ($n_{analyte}$ = 15, n_{sample} = 9, 4 out of 15 compounds were saturated, i.e. No. 12, 13, 15 and 16 labelled with an asterisk), MobT HS ($n_{analyte}$ = 6, n_{sample} = 17), and MobT DI ($n_{analyte}$ = 9, n_{sample} = 2) were organized and colored (light

to dark gray) according to the elution order in the stationary system. Terms' labels: "$n_{analyte}$" = number of identified compounds and "n_{sample}" = number of replicates.

S.4 Selective mass traces for peak identification and integration of all components of the standard mixture

Table S.4 Selective mass traces for peak identification and integration of all components of the standard mixture. Four mass traces were chosen per analyte: one ion for quantitation (denoted as "*quan ion*" and labeled as "m/z_1") and three identify-confirming ions (labeled as "m/z_2", "m/z_3" and "m/z_4") and organized by their descending relative abundances in the mass spectrum. Analytes were organized by elution order in the Stationary. "No." = designated ID number for each compound and "n.d." = not detected.

**No.**	**Compound**	**Retention time [min]**				**Selective mass traces**			
		**Stationary**	**MobE**	**MobH**[a]	**MobT**	m/z_1	m/z_2	m/z_3	m/z_4
**1**	Propan-2-one	2.3	n.d.	n.d.	n.d.	*43*	*58*	*42*	*44*[b]
**2**	*N*-Ethylethanamine	2.6	n.d.	0.9[c]	n.d.[d]	*58*	*44*	*73*	*72*
**3**	Hexane	n.d.	n.d.	1.2	n.d.	*57*	*56*	*86*	*71*
**4**	Butan-2-ol	2.6	n.d.	1.1	n.d.	*45*	*31*	*59*	*43*
**5**	Chloroform	2.7	3.3	n.d.	0.3[e]	*83*	*85*	*47*	*87*
**6**	1,2,3,4,5-Pentafluorobenzene	2.8	3.7	1.3	0.3[e,f]	*168*	*99*	*137*	*117*
**7**	Benzene	2.9	4.5	1.5	0.4[e]	*78*	*77*	*51*	*52*
**8**	Cyclohexane	3.0	n.d.	1.5	0.4[e]	*56*	*84*	*69*	*55*
**9**	Pyridine	3.4	7.3	2.0	0.7[f]	*79*	*52*	*51*	*78*
**10**	Toluene	3.7	7.6	2.4	0.5[e,f]	*91*	*92*	*65*	*51*
**11**	Hexan-1-ol	4.7	10.9	3.7	n.d.	*56*	*43*	*55*	*69*
**12**	1,2-Xylene	5.1	11.8	4.3	0.8[e,f]	*91*	*106*	*105*	*77*
**13**	Nonane	5.3	12.3	4.6	-[g]	*43*	*57*	*85*	*71*
**14**	Aniline	6.1	14.3	5.6	0.9[f]	*93*	*66*	*65*	*92*
**15**	Phenol	6.2	14.4	5.7	0.9[f]	*94*	*66*	*65*	*39*
**16**	4-Chlorophenol	9.4	19.5	8.9	1.2[f]	*128*	*65*	*130*	*100*
**17**	2,4,5-Trichlorophenol	11.7	22.7	10.4	1.4[f]	*196*	*198*	*97*	*200*
**18**	3,4-Dichlorophenol	12.4	23.4	10.8	1.5[f]	*162*	*164*	*99*	*63*

[a] Retention times were transformed from the original data format [h:min:s] to [min].

[b] Relative abundance [%] of the ion *m/z* 44 for propan-2-one could not be retrieved from the MAINLIB, NIST library.

[c] *N*-ethylethanamine in the MobH was identified by the vendor´s software, but it could not be identified and quantified by manual evaluation.

[d] *N*-ethylethanamine in the MobT was identified by longer extraction time of the fiber to the head-space of the closed-chamber vial, the rest of conditions were kept the same.

[e] Detected compounds in vapor state by the fiber exposed on the head-space (HS) of a closed chamber (9.1 mL total volume glass vial) containing 0.4 mL standard solution for 0.5 min extraction time.

[f] Detected compounds by direct immersion (DI) of the fiber in the standard solution (0.4 mL) for 0.5 min extraction time.

[g] An abundant peak eluting at the retention time corresponding most likely to nonane could not be identified by comparison with the NIST library in MobT because of a different base peak. The eluting peak was not considered for any estimation.

Note: For some compounds, different ions were selected in mobile devices compared with the ones from the Stationary as it is described in the following:

Device: compound (*selected ions m/z*)

MobE: nonane (*57, 85, 71, 128*), hexan-1-ol (*56, 55, 69, 84*), phenol (*94, 66, 65, 55*).

MobH: hexane (*57, 56, 86, 71*), phenol (*94, 66, 65, 55*), butan-2-ol (*45, 59, 74*).

MobT: toluene (*91, 92, 65, 63*), pyridine (*79, 52, 50, 78*), phenol (*94, 66, 65, 55*).

S.5 ANOVA on the effect of boiling point on the maximum-normalized response

An ANOVA was used to test the effect of the boiling point (explanatory variable) on the maximum-normalized response (response variable) of all identified analytes for each device (Stationary, MobE and MobH; refer to "Signal response patterns of the VOC standard mixture after TD/GC-MS analysis differ between the evaluated instruments" in the main manuscript). Normal distribution of the log-transformed response variable was tested with a Lilliefors (Kolmogorov-Smirnov) normality test, and the results showed normal distribution (p-value > 0.05) for each tested device. Results from the ANOVA are presented in terms of F-value and p-value for the explanatory variable. Significance was defined as p-value < 0.05.

Table S.5 Results of an ANOVA testing the effect of boiling point (at standard pressure, a proxy of volatility) on the maximum-normalized response of the *quan ion* of all identified analytes (in logarithmic scale) (response pattern) of the stationary device ($n_{analyte}$ = 17, n_{ion} = 17, n_{sample} = 7) and the two portable systems: Mob E ($n_{analyte}$ = 13, n_{ion} = 13, n_{sample} = 3) and MobH ($n_{analyte}$ = 11 out 15, n_{ion} = 11 out 15, n_{sample} = 9, 4 detected analytes were not considered due to saturation of the base peak). Labels' terms: "$n_{analyte}$" = number of identified compounds, "n_{ion}" = number of selective ions (base peak, in this case) from all identified analytes and "n_{sample}" = number of replicates. Significant *p*-values are labelled in bold.

**Factor**	**$\log_{10}$ (Maximum-normalized signal response)**					
	**Stationary**		**MobE**		**MobH**	
	***F*-value**	***p*-value**	***F*-value**	***p*-value**	***F*-value**	***p*-value**
Boiling point (b.p.)	0.6	0.47	3.1	0.11	9.7	**0.01**
$n_{analyte}$, n_{ion}, n_{sample}	17, 17, 7		13, 13, 3		11, 11, 9	

S.6 Ordinary least squares regression analyses of analytical parameters for stationary and mobile GC-MS devices

Table S.6 describes the results from the ordinary least squares regression analyses evaluating the effect of the **explanatory variables (EV):** (i) mass of the fragment, (ii) fragment's absolute abundance (*i.e.* the intensity of each ion to the maximal abundant peak from all analytes from a particular instrument) and their interaction on the following **response variables (RV)**: (i) % RSD (refer to "Mobile instruments exhibit a poorer mass spectral reproducibility"); (ii) % absolute error (refer to "Mobile instruments exhibit a poorer mass spectral similarity for identification with mass spectral libraries"); and (iii) *S/N* (refer to "Mobile instruments exhibit a lower sensitivity") for each instrument individually. Normal distribution of each log-transformed response variables was tested with a Lilliefors (Kolmogorov-Smirnov) normality test, and the results showed normal distribution in all cases (p-value > 0.05).

Table S.6 Results from the ordinary least squares regression analyses evaluating the effect of the **explanatory variables (EV):** (i) mass of the fragment, (ii) fragment's absolute abundance and their interaction on the following **response variables (RV)**: (i) % RSD; (ii) % absolute error; and (iii) *S/N* for each instrument individually. Fragments with values above 30 % RSD were not considered for the linear models. Terms' labels: "*b*" = intercept, "*m*" = slope, *p*-value, "$n_{analyte}$" = number of detected analytes, "n_{ion}" = number of selective ions from all detected analytes, "n_{sample}" = replicates, and "n.a." = not available values. For significant linear correlations, bold font is used.

Parameter (RV)	Terms	Stationary	MobE	MobH	MobT
		EV: log_{10} (m/z)			
Reproducibility (log_{10} %RSD)	*B*	1.18	1.41	1.58	1.31
	M	-0.40	-0.26	-0.54	-0.19
	p-value	0.10	0.38	0.11	0.63
	$n_{analyte}$,n_{ion},n_{sample}	17,50,7	13,36,3	15,41,9	6,18,17[a]; 9,27,2[b]
Similarity (log_{10} (Absolute error x 100))	*B*	**2.81**	2.62	**4.44**	2.68
	M	**-0.95**	-0.73	**-0.62**	-0.65
	p-value	**0.03**	0.05	**<0.001**	0.08
	$n_{analyte}$,n_{ion},n_{sample}	17,49,7	13,33,3	15,37,9	6,18,17[a]; 9,27,2[b]
Signal-to-noise ratio (log_{10} S/N)	*B*	**-7.25**	**-1.28**	n.a.	**-3.35**
	M	**5.31**	**1.62**	n.a.	**2.84**
	p-value	**< *0.001***	**<0.01**	n.a.	**<0.001**
	$n_{analyte}$,n_{ion},n_{sample}	11,44,7	11,36,3	n.a.	5,20,17[a]; 9,27,2[b]
		EV: log_{10} (Absolute abundance)			
Reproducibility (log_{10} %RSD)	*B*	**0.70**	**1.25**	0.51	1.01
	M	**-0.27**	**-0.32**	0.04	-0.12
	p-value	**<0.01**	**0.01**	0.70	0.29

	$n_{analyte},n_{ion},n_{sample}$	17,50,7	13,36,3	15,41,9	6,18,17[a]; 9,27,2[b]
Similarity (log$_{10}$ (Absolute error x 100))	*B*	1.17	**1.77**	**1.72**	1.45
	M	0.14	**-0.54**	**-0.36**	-0.04
	p-value	0.48	**<0.01**	**0.049**	0.70
	$n_{analyte},n_{ion},n_{sample}$	17,49,7	13,33,3	**15,37,9**	6,18,17[a]; 9,27,2[b]
Signal-to-noise ratio (log$_{10}$ S/N)	*B*	**1.62**	**0.55**	n.a.	**1.44**
	M	**0.88**	**1.06**	n.a.	**0.92**
	p-value	**0.04**	**<** ***0.001***	n.a.	**<0.001**
	$n_{analyte},n_{ion},n_{sample}$	11,44,7	11,36,3	n.a.	5,20,17[a]; 9,27,2[b]

[a] Data obtained by HS-SPME with a PDMS-DVB fiber, MobT HS.
[b] Data obtained by DI-SPME with a PDMS-DVB fiber, MobT DI.
Note: raw data MobH had to be excluded from the evaluation of signal-to-noise ratio to keep the orthogonality in the comparison (import as "*.cdf*" files were not feasible); therefore, "n.a." is used for not available estimation.

S.7 RSD of signal response in dependence on the *mass* and *absolute abundance* of the fragments in a linear regression for the stationary and mobile systems

To identify the reason for the poor precision, % RSD of all evaluated fragments as a function of specific independent variables was related to **(a)** the *m/z* (mass) of the fragment and **(b)** the fragment's absolute abundance in a linear regression (see Supp. Table S.6 for tabulated results and the following Fig. S.7 for illustration). As expected from the already estimated ratio between mean %RSD values (refer to Table 3), the mass spectral variability was found significantly reduced for high abundant ions (negative slope, *m*) in both types of devices (***p*-value = 0.01**, *m* = -0.32 and *b* = 1.25 for the portable MobE and ***p*-value < 0.01**, *m* = -0.27 and *b* = 0.70 for the stationary system). The better performance, *i.e.* better reproducibility and dynamic range of the Stationary (seen in Table 3) is represented here through a higher significance, smaller slope and intercept (***m* = -0.27** and ***b* = 0.70**) in the linear correlation between % RSD and the [absolute intensity]. In addition, a shorter range in the x axis (range of abundance) for the quadrupole mass analyzers (portable and stationary devices) (Fig. S.7b) indicates lower selectivity of these devices, a benefit when multi-selective analyses are anticipated. Indeed, the worse performance of the portable devices (with high RSD values according to Table 3) such as MobT is also represented by the larger difference in signal response between the least and most abundant fragments, which extends the range of the x axis and suggests a higher fragment selectivity of this device. The latter may also be a disadvantage for the identification of volatiles by spectra comparison with customer-reference libraries.

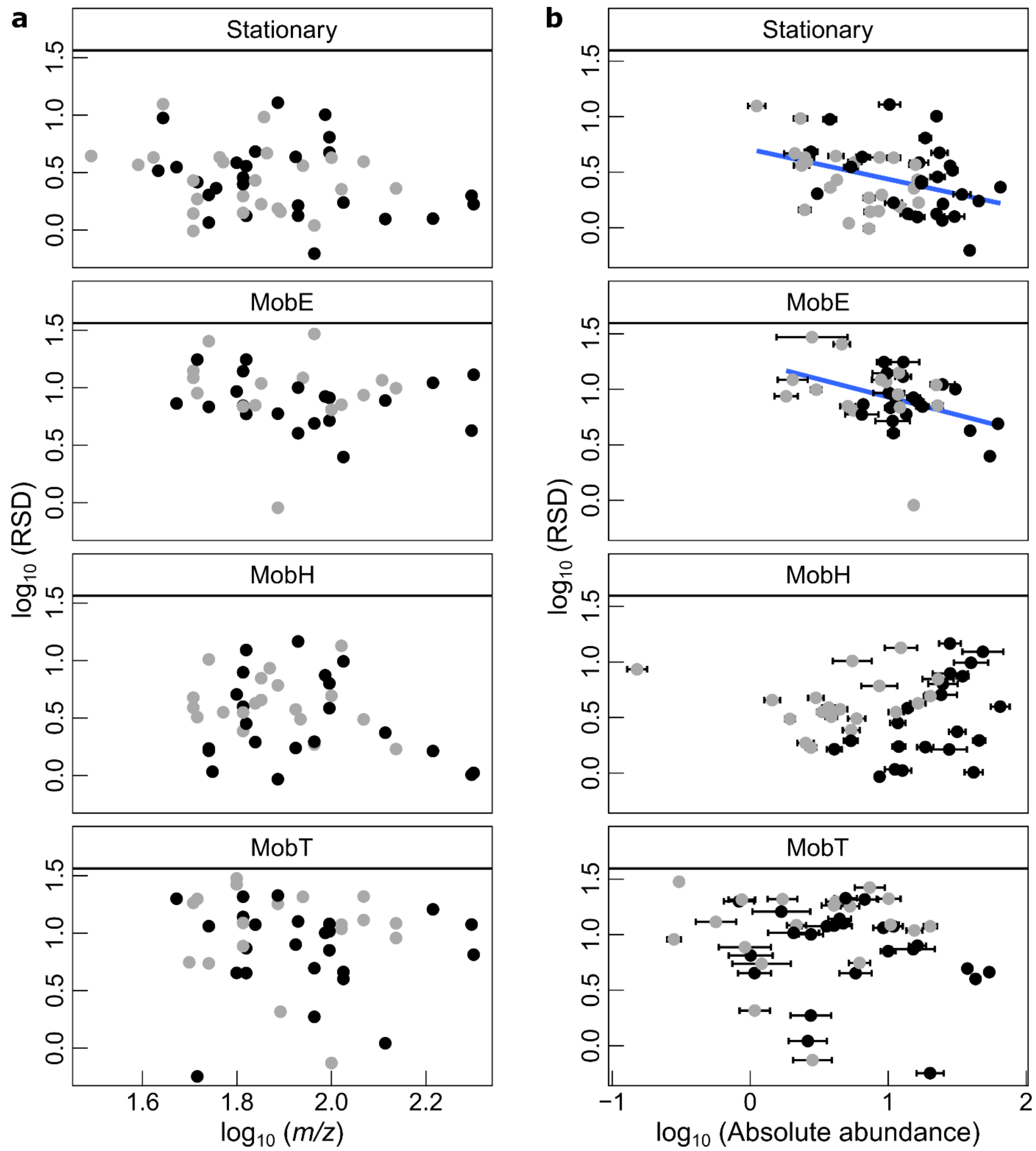

Fig. S.7 Ggplots representing the % RSD from the observed relative abundance of replicate analyses of three selective mass traces per analyte plotted over (a) their corresponding *m/z* or (b) the fragment's absolute abundance for the Stationary ($n_{analyte}$ = 17, n_{ion} = 50, n_{sample} = 7, except for butan-2-ol *m/z* 43) and the mobile GC-MS systems (MobE, $n_{analyte}$ = 13, n_{ion} = 36, n_{sample} = 3, except for pyridine *m/z* 78,51 and aniline m/z 65; MobH, $n_{analyte}$ = 15, n_{ion} = 41, n_{sample} = 9, except for butan-2-ol *m/z* 43 and pyridine; MobT HS, $n_{analyte}$ = 6, n_{ion} = 18, n_{sample} = 17; and MobT DI, $n_{analyte}$ = 9, n_{ion} = 27, n_{sample} = 2). Terms' labels: "$n_{analyte}$" = number of identified analytes, "n_{ion}" = number of selective ions of a particular abundance range and "n_{sample}" = number of replicates. Dark grey dots illustrate fragments with $a_1 \leq 25\%$ and black dots with $a_2 > 25\%$, where "a" is relative abundance; blue lines represent significant linear correlations upon occurrence. (Note: ions with % RSD higher than 30% were considered as outliers and therefore excluded from the statistical analysis and illustration – relates to <10% of the total values.) Results from the linear regression analysis are tabulated in the supplementary section (Supp. S.6, Table S.6).

S.8 Exemplary spectra comparison between stationary and mobile GC-MS devices - phenol

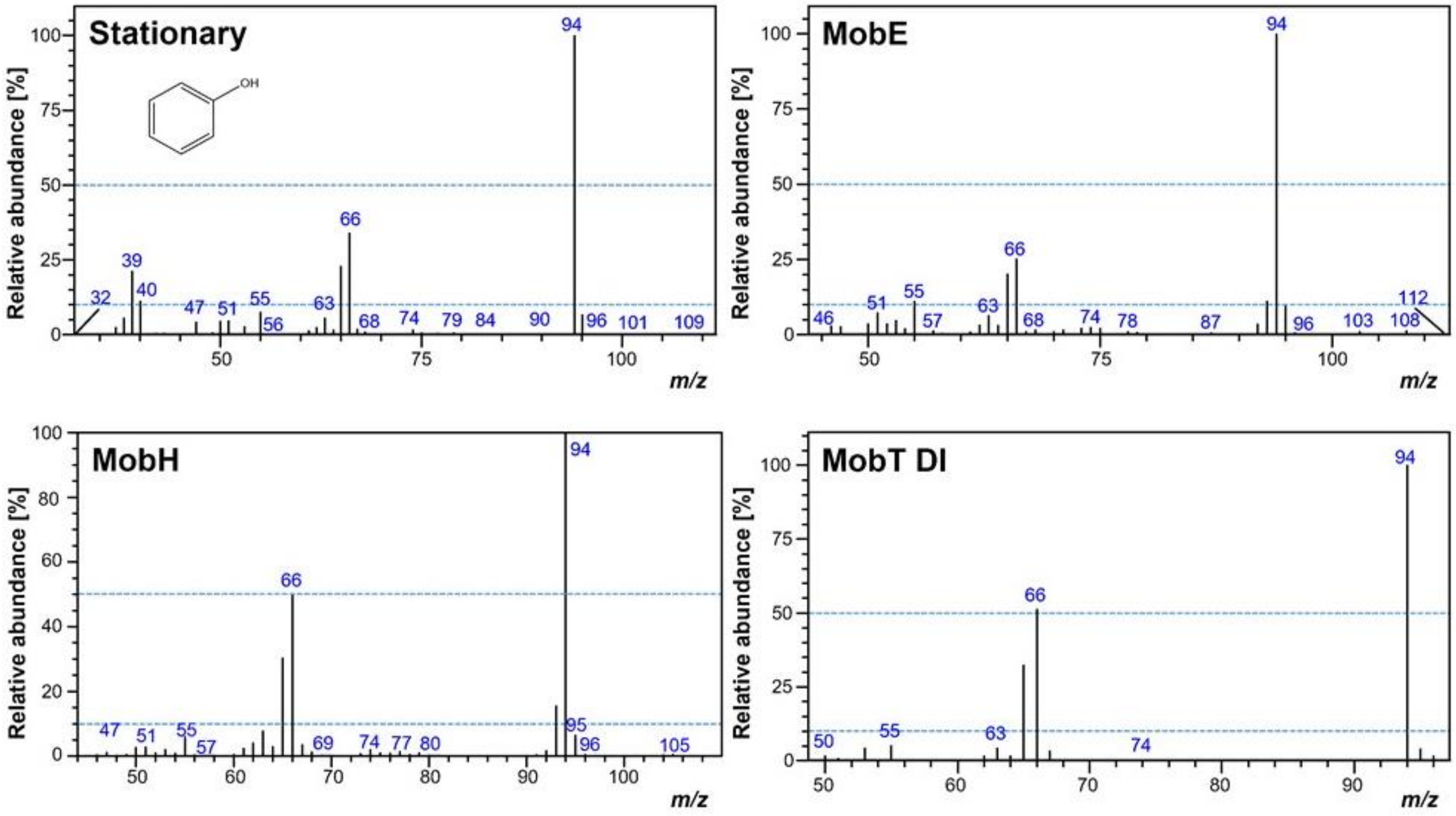

Fig. S.8 Comparison of the spectra (scan at the apex of the peak) of the aromatic compound, phenol from the Stationary, MobE and MobT (HS or DI) using GCMS solution 4.20 software (Shimadzu, Kyoto, Japan). For MobH, the spectrum was illustrated by ER IQ software version 2.33 (Inficon, Inc., Germany) and formatted. Blue dashed lines have been arbitrarily added to limit 10 and 50% relative abundance and ease the comparison.

S.9 Signal-to-noise ratio (*S/N*) for stationary and mobile TD/GC-MS devices

Table S.9 Signal-to-noise ratio (*S/N*) estimated by RMS method for analytes identified in the Stationary ($S/N_{Stat.}$, $n_{analyte}$ = 11, n_{ion} = 21, n_{sample} = 7, no toluene *m/z* 92) and MobE (S/N_{MobE}, $n_{analyte}$ = 11, n_{ion} = 20, n_{sample} = 3, except for pentafluorobenzene *m/z* 168, nonane *m/z* 43) for the two most abundant selective mass traces per analyte. *S/N* values corresponded to the total ng of analyte spiked in the thermal desorption tube (refer to Table 1). *S/N* values were categorized in four ranges and colored as following: 1 – 10 (“dark gray”), > 10 – 100 (“gray”), > 100 – 1000 (“medium gray”) and > 1000 (“light gray”). “No.” = designated ID number for each compound, “SD” = standard deviation, “% RSD” = relative standard deviation between replicates and “n.a.” = not available.

No.	Compound	*m/z*	*S/N* (RMS method, per total ng/tube[c]) Instrument ($n_{analyte}$, n_{ion}, n_{sample})						S/N_{MobE} / $S/N_{Stat.}$ (x1000)
			Stationary (11, 21, 7)			MobE (11, 20, 3)			
			Mean	SD	%RSD	Mean	SD	%RSD	
5	Chloroform	*83*	11826	3931	33	54	1	3	5
		85	13832	1972	14	47	4	8	3
6	1,2,3,4,5-Pentafluorobenzene	*168*[a]	90406	30801	34	n.a.	n.a.	n.a.	
		99	33580	7123	21	66	11	17	2
7	Benzene	*78*	1151	147	13	149	19	13	130
		77	389	65	17	53	18	34	137
9	Pyridine	*79*	230	17	7	48	5	10	207
		52	35	2	5	22	6	29	609
10	Toluene	*91*	697	54	8	388	94	24	556
		92	n.a.	n.a.	n.a.	412	159	39	
11	Hexan-1-ol	*56*	1040	398	38	71	12	17	68
		43, 84[b]	159	68	43	5	2	44	34
12	1,2-Xylene	*91*	1681	417	25	410	101	25	244
		106	14420	8583	60	327	32	10	23
13	Nonane	*43*[b]	680	371	55	n.a.	n.a.	n.a.	
		57	888	638	72	265	96	36	299
14	Aniline	*93*	884	167	19	132	14	10	150
		66	212	23	11	29	10	33	135
15	Phenol	*94*	20921	9430	45	234	50	21	11
		66	264	15	6	55	15	28	208
16	4-Chlorophenol	*128*	35557	15385	43	732	32	4	21
		65	2602	392	15	73	10	13	28
		Maximum	**90406**			**732**			**609**
		Minimum	**35**			**5**			**2**
		Median	**1040**			**72**			**130**
		Mean	**11022**			**179**			**151**

[a] *S/N* for 1,2,3,4,5-Pentafluorobenzene by the RMS calculation method for the MobE could not be determined by the software.
[b] Different ions were selected between mobile and stationary devices due to applied different mass ranges. For hexan-1-ol *m/z* 43 and 84, for Stationary and MobE, respectively. For nonane, *m/z* 43 could not be analyzed with MobE.
[c] Total ng of compound injected per tube can be seen in Table 1.

S.10 Signal-to-noise ratio (*S/N*) in dependence on the boiling point

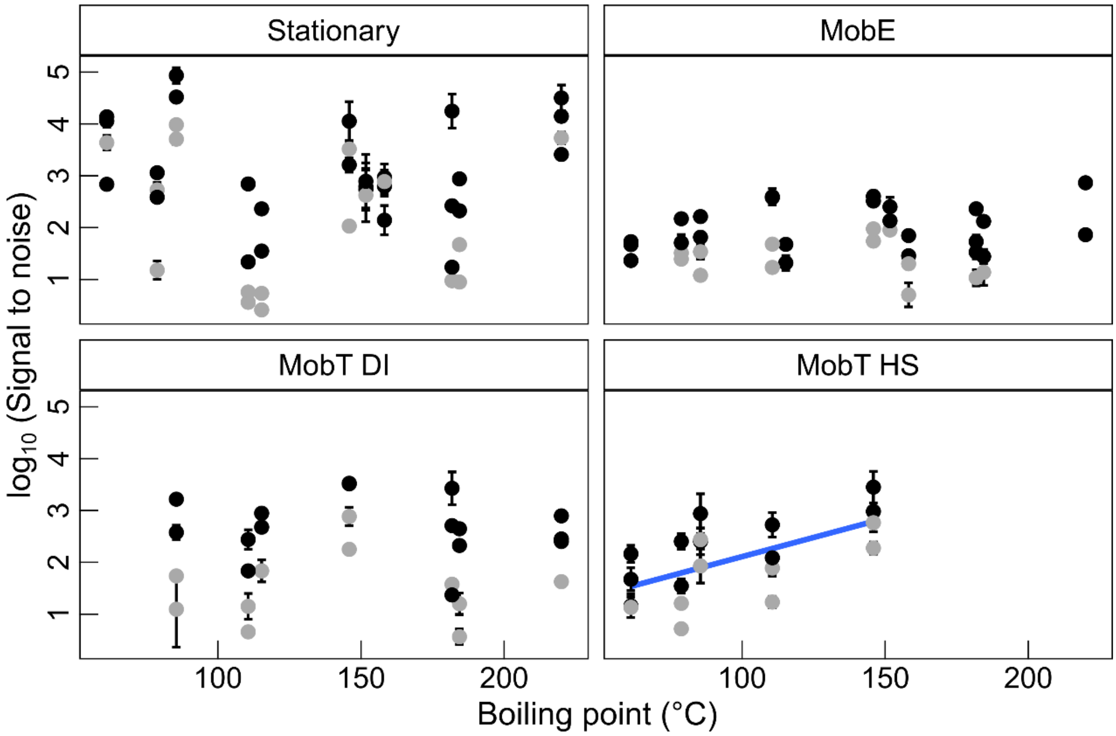

Fig. S.10 *S/N* (RMS calculation) of four selective mass traces of each compound detected by the Stationary compared to the MobE and MobT in logaritmic scale, distributed according to their respective *boiling point (as a proxy of volatility)* for all evaluated analytes. Representative analytes commonly detected in the Stationary ($n_{analyte}$ = 11, n_{ion} = 44, n_{sample} = 7), MobE ($n_{analyte}$ = 11, n_{ion} = 36, $n_{replicate}$ = 3, except for chloroform *m/z* 87, pentafluorobenzene *m/z* 168, piridyne *m/z* 51, 78, nonane *m/z* 128, aniline *m/z* 65, 4-chlorophenol *m/z* 130, 100), MobT DI ($n_{analyte}$ = 9, n_{ion} = 27, n_{sample} = 2) and MobT HS ($n_{analyte}$ = 5, n_{ion} = 20, n_{sample} = 17) were used for illustration. Each *S/N* value is colored in dark grey dots for $a_1 \leq 25$ and black dots for $a_2 > 25$, where "a" is abundance. Terms labels: "$n_{analyte}$" = number of detected analytes, "n_{ion}" = number of selective ions per particular abundance range and "n_{sample}" = number of replicates. Significant linear correlations were not found, except for MobT HS. (Note: particularly for MobE, the *S/N* for very low abundant ions could not be calculated through RMS by the applied software, but PtoP.)

S.11 RSD from the observed relative abundance of replicate analyses of selective mass traces for a stationary GC-MS device compared to portable systems

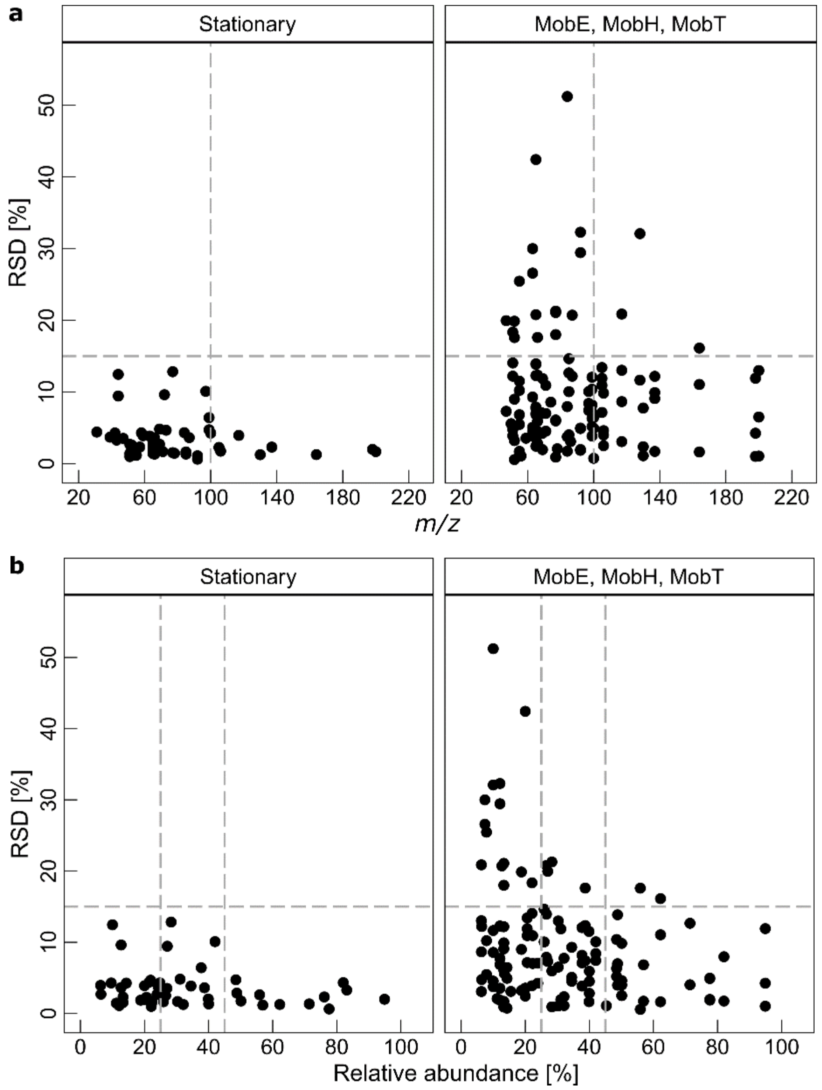

Fig. S.11 Ggplots representing the % RSD from the relative abundance of replicate analyses of three selective mass traces per analyte plotted over **(a)** their corresponding *m/z* or **(b)** the fragment's relative abundance for the Stationary ($n_{analyte}$ = 17, n_{ion} = 50, n_{sample} = 7, except for butan-2-ol *m/z* 43) compared to the mobile GC-MS systems (MobE, $n_{analyte}$ = 13, n_{ion} = 36, n_{sample} = 3, except for pyridine *m/z* 78,51 and aniline *m/z* 65; MobH, $n_{analyte}$ = 15, n_{ion} = 41, n_{sample} = 9, except for butan-2-ol *m/z* 43 and pyridine; MobT HS, $n_{analyte}$ = 6, n_{ion} = 18, n_{sample} = 17; and MobT DI, $n_{analyte}$ = 9, n_{ion} = 27, n_{sample} = 2). Terms' labels: "$n_{analyte}$" = number of identified analytes, "n_{ion}" = number of selective ions of a particular abundance range and "n_{sample}" = number of replicates. Dashed grey lines cutting the y axis limit the maximum %RSD values obtained in the Stationary. Dashed grey lines cutting the x axis were arbitrarily added to limit the variance of ions with relative abundances below 25% (already explained before) and 45% (based on the higher frequency of RSD values above the maximum in the y axis).

S.12 Mass spectral similarity in terms of % absolute error from selective mass traces for the stationary device compared to portable GC-MS systems

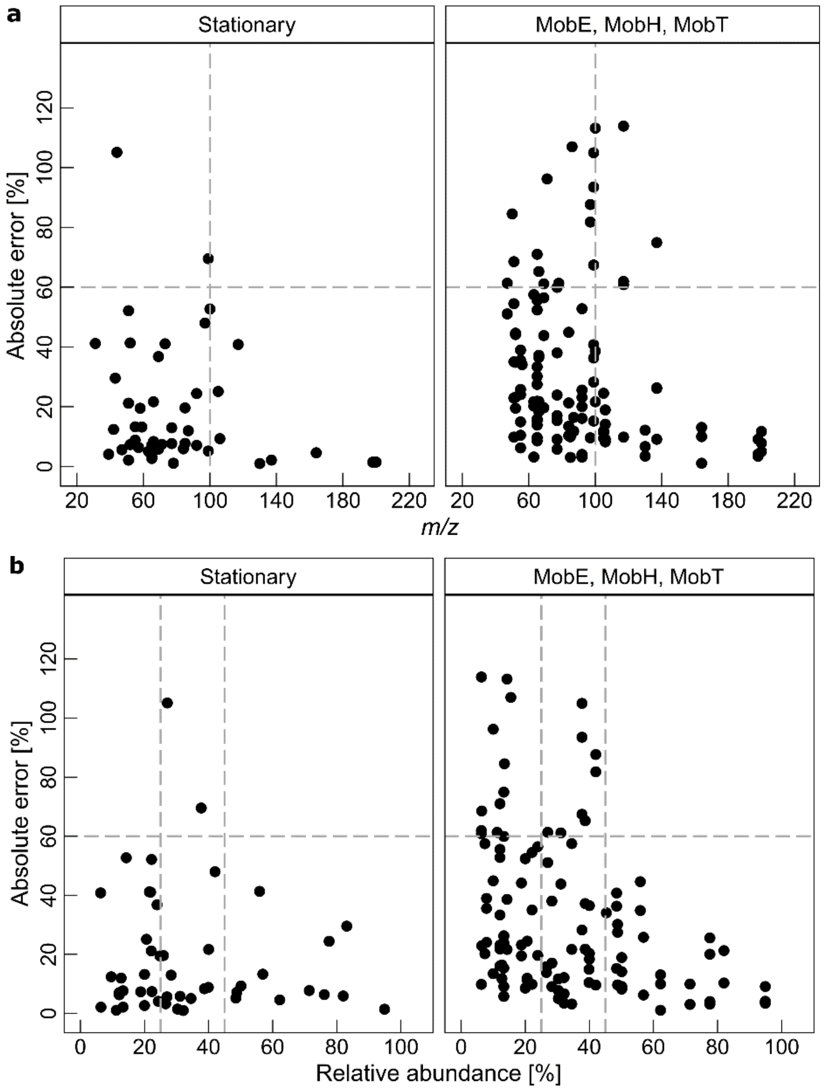

Fig. S.12 Ggplots representing the mass spectral similarity in terms of % absolute error from each selective ion plotted over **(a)** their corresponding *m/z* or **(b)** the fragment's relative abundance for the Stationary ($n_{analyte}$ = 17, n_{ion} = 49, n_{sample} = 7, except for propan-2-one *m/z* 44 and butan-2-ol *m/z* 43), MobE ($n_{analyte}$ = 13, n_{ion} = 33, n_{sample} = 3, except for pyridine *m/z* 78, 51, aniline *m/z* 65 and nonane* with filtered base peak), MobH ($n_{analyte}$ = 15, n_{ion} = 37, n_{sample} = 9, only butan-2-ol *m/z* 59, except for nonane with filtered base peak* and pyridine), MobT HS ($n_{analyte}$ = 6, n_{ion} = 18 , n_{sample} = 17), and MobT DI ($n_{analyte}$ = 9, n_{ion} = 27, n_{sample} = 2). Terms' labels: "$n_{analyte}$" = number of identified analytes, "n_{ion}" = number of selective ions from all identified analytes in a particular abundance range and "n_{sample}" = number of replicates. *Nonane could not be compared with the NIST library since the base peak *m/z* 43 was below the adjusted mass range in MobE and MobH (>*m/z* 45). Dashed grey lines in the y axis delimited the maximum values of % absolute error obtained in the Stationary. Dashed grey lines cutting the x axis were arbitrarily added to limit the variance of ions with relative abundances below 25% (already explained before) and 45% (based on the higher frequency of RSD values above the maximum in the Stationary).

References

1. Mielczarek P, Silberring J, Smoluch M. Miniaturization in mass spectrometry. Mass Spectrom Rev. 2020; https://doi.org/10.1002/mas.21614
2. Inficon Inc. Operating manual: HAPSITE ER chemical identification system. IPN 074-471-P1B. 2009.
